# Intracerebroventricular calycosin attenuates cerebral ischemia-reperfusion injury in rats via HMGB1-dependent pyroptosis inhibition

**DOI:** 10.3389/fphar.2025.1596087

**Published:** 2025-06-18

**Authors:** Yuhui Zhang, Qiguang Wu, Yanjin Pan, Min Xin, Wenbo Wang, Xiaoya Zhai, Peiquan Zhou, Chong Zhang, Yong Wang

**Affiliations:** ^1^ Key Laboratory of Tumor Immunology and Microenvironmental Regulation, Guilin Medical University, Guilin, China; ^2^ Department of Physiology, Guilin Medical University, Guilin, China; ^3^ Department of Neurosurgery, Nanxishan Hospital of Guangxi Zhuang Autonomous Region, Guilin, China; ^4^ Laboratory of Neurological Diseases, Nanxishan Hospital of Guangxi Zhuang Autonomous Region, Guilin, China; ^5^ Department of Neurosurgery, Affiliated Hospital of Guilin Medical University, Guilin, China; ^6^ Department of Neurology, The Second Affiliated Hospital of Guilin Medical University, Guilin, China; ^7^ Guangxi Medical and Health Key Cultivation Discipline Construction Project, Guilin, China

**Keywords:** cerebral ischemia-reperfusion injury, calycosin, neuroprotection, pyroptosis, HMGB1, NLRP3

## Abstract

**Introduction:**

HMGB1-NLRP3 mediated pyroptosis was recently discovered to be a pathogenic cause of ischemic stroke. Our previous research has demonstrated the anti-inflammatory and anti-apoptotic properties of calycosin in mitigating cerebral ischemia-reperfusion injury (CIRI). However, its specific effects on HMGB1-NLRP3-mediated pyroptosis in ischemic stroke remain unclear. This study investigated the efficacy of calycosin in reducing pyroptosis-linked CIRI.

**Methods:**

*In vivo*, a rat model of middle cerebral artery occlusion (MCAO) received varying doses of intracerebroventricular calycosin. Therapeutic efficacy was assessed using neurological deficit scores, TTC staining, H-E staining, Nissl staining, and immunohistochemistry. *In vitro*, HAPI microglial cells were subjected to oxygen-glucose deprivation/reoxygenation (OGD/R) and then treated with calycosin, HMGB1 siRNA, or MCC950. Cell survival was evaluated using the CCK8 assay. Ultrastructural changes were examined through transmission and scanning electron microscopy. Inflammatory cytokine levels were quantified by ELISA. The expression of pyroptosis-related proteins and genes was analyzed using Western blot and qRT-PCR.

**Results and Discussion:**

Calycosin significantly reduced neurological impairments and brain infarction in a dose-dependent manner, alleviated neuronal damage and decreased the expression of pyroptosis-related markers, including NLRP3, GSDMD, HMGB1, IL-1β, IL-18, and caspase-1. These results indicate that calycosin enhances microglial cell survival and mitigates pyroptotic damage by inhibiting NLRP3 inflammasome activation, suggesting its potential as a neuroprotective therapy for ischemic stroke through the modulation of the HMGB1-dependent pyroptosis pathway.

## 1 Introduction

Ischemic stroke (IS), a major cause of mortality and impairment globally, occurs when the brain’s blood flow is disrupted (Feigin et al., 2023). Rapid restoration of blood flow is the primary goal of acute stroke treatment, as it can salvage the ischemic penumbra and minimize infarct expansion (Jadhav and Campbell, 2021). However, if the ischemia persists for too long (usually more than 4 h), the restoration of blood supply (reperfusion) itself can exacerbate brain injury through a complex cascade of pathological mechanisms collectively termed cerebral ischemia-reperfusion injury (CIRI) (Ma et al., 2021b). As an intricate program, CIRI involves numerous molecular and cellular processes, including inflammation, oxidative stress, excitotoxicity, and apoptosis (Wang et al., 2023; Yu et al., 2023; Li et al., 2024b). Understanding the underlying molecular causes of CIRI may help develop effective neuroprotective therapies.

In recent years, a unique type of programmed cell death, pyroptosis, has attracted attention for its role in inflammatory disorders, including ischemic stroke (Franke et al., 2021; Coll et al., 2022; Li et al., 2023a). Pyroptosis is mainly driven by the formation of the NLRP3 inflammasome, an array of proteins that triggers caspase1 and eventually leads to the maturation and secretion of IL-1β and IL-18 (Ma, 2023). High mobility group box 1 (HMGB1), an outer-cell-released and unique nuclear protein that functions as the damage-associated molecular pattern (DAMP), can be detected by the NLRP3 inflammasome (Ratajczak et al., 2019; Li et al., 2024a). Research has linked the HMGB1-NLRP3 route to the development of CIRI, with HMGB1 enhancing NLRP3 inflammasome activation, which in turn escalates pyroptotic cell death and inflammatory responses (Ye et al., 2019; Liao et al., 2024; Zhang et al., 2024b). Meanwhile, this inflammation cascade occurring during the ischemic and reperfusion phases is closely related to excessive microglial polarization (Liu et al., 2017; Ma et al., 2021a; Zhang et al., 2024a). Microglia play a double-edged role in CIRI (Hernandez et al., 2021). On one hand, they exhibit protective functions, such as phagocytosing pathogens and facilitating tissue repair. On the other hand, their excessive polarization leads to the production of pro-inflammatory factors and neurotoxins, triggering an inflammatory cascade that contributes to CIRI. Therefore, inhibiting excessive microglial polarization may be a potential therapeutic strategy for stroke (Kumari et al., 2024; Zeng et al., 2024).

*Astragali Radix* (AR), originally recorded in Shennong’s herbal, is widely utilized in the prevention and treatment of clinical cardiovascular and cerebrovascular disorders (Tan et al., 2020; Li et al., 2023c). As a traditional Chinese medicinal herb, the renowned therapeutic properties of AR on immune modulation, Qi tonification, blood nourishment, and diuretic effects are utilized for ischemic stroke (Liu et al., 2016; Li et al., 2020). Calycosin, a biologically active isoflavonoid derived from AR (Deng et al., 2021), has demonstrated neuroprotective potential in CIRI through anti-oxidation, anti-calcium overload, anti-ferroptosis, and suppression of microglial overactivation (Yan et al., 2019; Hsu et al., 2020; Liu et al., 2023; Xu et al., 2023). Emerging evidence indicates that calycosin inhibits NF-κB pathway activation and reduces inflammatory mediator release by blocking HMGB1 secretion and its binding to downstream receptors. In models of severe acute pancreatitis and sepsis, calycosin downregulates HMGB1 expression, suppresses NF-κB p65 phosphorylation and MyD88/NF-κB signaling, and alleviates histopathological damage (Chen et al., 2021; Zhu et al., 2021a). Moreover, through inhibition of mitochondrial ROS generation and NLRP3 inflammasome activation, calycosin diminishes pro-inflammatory cytokines IL-1β and IL-18, demonstrating protective effects in sepsis-induced acute lung injury and acute myocardial infarction (Xia et al., 2021; Yuan et al., 2025). These findings provide a scientific foundation for calycosin’s application in anti-inflammatory and immunomodulatory therapies, suggesting its potential as a novel strategy for prevention of ischemic stroke. Our previous research revealed that calycosin protects microglia from OGD/R-induced damage via HMGB1/TLR4/NF-κB pathway inhibition (Li et al., 2023b). However, whether its neuroprotective effects involve HMGB1-dependent pyroptosis requires further investigation. Building on our prior findings confirming its anti-neuroinflammatory, anti-autophagic, and anti-apoptotic properties (Wang et al., 2018; Li et al., 2023b), this study addresses a critical knowledge gap regarding HMGB1-dependent pyroptosis in CIRI pathogenesis and potential pharmacological modulation by calycosin.

The intracerebroventricular administration route offers distinct pharmacokinetic advantages for cerebral drug delivery. This approach utilizes cerebrospinal fluid (CSF) dynamics to bypass hepatic first-pass metabolism and the blood-brain barrier. As a result, therapeutic agents can be directly transported to ischemic lesions through ventricular conduits. Such targeted delivery ensures optimal central nervous system bioavailability while minimizing systemic exposure (Sadekar et al., 2022). Our experimental design involved prophylactic intracerebroventricular administration of calycosin to systematically evaluate its regulatory effects on the HMGB1-NLRP3 pyroptosis pathway in a rat CIRI model.

This research employed intracerebroventricular delivery in rats to explore the localized impact of calycosin on brain ischemia-reperfusion damage. Based on the HMGB1-NLRP3-mediated pyroptosis pathway, we further investigated the underlying neuroprotective mechanisms of calycosin in this cerebral ischemia-reperfusion damage model.

## 2 Materials and methods

### 2.1 Animals and reagents

Hunan Slake Jingda Experimental Animal Co., Ltd. in Changsha, China, is the supplier of male SPF-grade Sprague-Dawley rats weighing between 220 and 280 g. The rodents at the Guilin Medical University Animal Center were kept in a controlled environment with a constant supply of food and water, a 12-h light/dark cycle, 40%–60% humidity, and 24°C ± 2°C. The Experimental Protocol was authorized by Guilin Medical University’s Ethics Committee and followed their animal care criteria.

Calycosin (C_16_H_12_O_5_, purity >98%; B9938) and LPS (Lipopolysaccharide, L4391) were bought from MO, United States’s Sigma-Aldrich. MCC950 (a specific NLRP3 inhibitor, KM3631, KKL Med Inc., United States) and Nigericin (a NLRP3 activator, nigericin sodium salt, KM4757, KKL Med Inc., United States) were obtained from KKL Med Inc.

For intracerebroventricular administration, 5, 1.25, and 2.5 mg/mL solutions were made from the 500 mg/mL stock solution that was created by dissolving calycosin in DMSO (D8370, Solarbio, Beijing, China). For cell culture, a 0.1 M stock solution was created by dissolving calycosin in DMSO. Before use, it was diluted with culture medium to achieve the necessary final concentrations.

Solutions of LPS, MCC950, and Nigericin were made following the guidelines provided by the manufacturer. In detail, LPS was mixed with PBS to make a stock solution of 2 mg/mL, then diluted with culture medium to 1 μg/mL before application. MCC950 and Nigericin were mixed in DMSO to create 5 mM stock solutions, then diluted with culture medium to 10 μM before use. These reagents were kept at 4°C.

### 2.2 Animal grouping and intracerebroventricular injection

One MCAO group, three treatment groups (containing 75, 150, and 300 μg/kg of calycosin), and a sham-operated group were among the 80 rats that were distributed at random to these groups. Calycosin or vehicle (1% DMSO +99% saline for both the sham-operated and MCAO groups) was administered intracerebroventricularly at equivalent volumes.

Following a week-long adjustment phase, the rats were anesthetized intraperitoneally with a 1% sodium pentobarbital (50 mg/kg) and gently fixed to the stereotaxic device (69,100, RWD, Shenzhen, China). Delivering calycosin or a vehicle with a rate of 1 μL/min entering the right lateral ventricle was done using a micro-injection pump (KDS LEGATO 130, RWD, Shenzhen, China) coupled to a 25 μL micro-syringe (Hamilton, Reno, NV). For the injection site, the stereotaxic coordinates were as follows: AP (Anterior-Posterior, Y-axis): 0.8 mm; ML (Medial-Lateral, X-axis): 1.5 mm; and DV (Dorsal-Ventral, Z-axis): 3.8 mm. Following the injection, the needle was gently removed after being in place for 15 min. The body temperature of the rats was kept at 37°C ± 0.5°C during the procedure.

### 2.3 MCAO model preparation

The MCAO model was established 24 h after the intracerebroventricular injection. After administering 1% sodium pentobarbital (50 mg/kg) intraperitoneally for anesthesia, make an incision at the midline in the neck region to expose and divide the right external carotid artery (ECA), internal carotid artery (ICA), and common carotid artery (CCA). A single-strand nylon suture (MSRC37B200PK50, RWD, Shenzhen, China) was threaded through CCA, pushed into ICA, and placed at the start of the middle cerebral artery until minor resistance was encountered, signifying successful blockage. The insertion depth was approximately 1.8–2.0 cm. After 1.5 h of blockage, it was carefully pulled out to allow circulation to resume. In the sham-operated group, no sutures were placed. The rats’ body temperature was maintained constant during the procedure.

A total of 80 male Sprague-Dawley rats were enrolled in this study, including 16 rats in the sham-operation group and 64 rats subjected to MCAO. After excluding 5 rats that died during the operation, 2 rats without neurological deficits, and 1 rat confirmed to have subarachnoid hemorrhage, 56 MCAO rats met the inclusion criteria. All surgical procedures were conducted by certified microvascular technicians. Subsequently, the eligible animals were randomly assigned to different experimental groups.

### 2.4 Neurological deficit assessment and TTC staining

An investigator blinded to the experimental groups assessed the neurological deficits in rats using the Zea-Longa scoring method after reperfusion (Longa et al., 1989). A total of 5 points, including 0 for normal behavior without neurological issues, 1 for inability to extend the left forepaw, 2 for leftward circling while crawling, 3 for falling to the left during crawling, and 4 for inability to walk on their own or loss of consciousness.

Slices of rat brain tissue, 2 mm thick, were subsequently incubated with a 1% TTC solution (T8170, Solarbio, Beijing, China) at 37°C for 15 min in the dark. After obtaining sections of brain tissue, the infarct volume was calculated with ImageJ software by adding up the damaged regions in each slice and then multiplying by the slice thickness. The ratio of cerebral infarct volume (%) was quantified with edema correction using the following formula (Wang et al., 2021; Hou et al., 2025):

$$\left[\left(\text{contralateral hemisphere volume}-\text{non}-\text{infarcted area volume of ipsilateral hemisphere}\right)\;/\text{ contralateral hemisphere volume}\right]\times 100\%.$$

### 2.5 Hematoxylin & Eosin (H-E) staining, Nissl staining and immunohistochemistry staining

To evaluate histopathological changes, paraffin-embedded brain samples were stained with H-E. The brains were stored in 4% paraformaldehyde for a day after euthanasia, then cleaned with xylene, dehydrated using a range of ethanol concentrations, and finally embedded in paraffin. After the slides were stained with hematoxylin and eosin (ZLI-9613, ZSGB-BIO, Beijing, China) and sealed, the pictures were taken under a light microscope.

Nissl staining was performed to assess neuronal damage and loss. Paraffin-embedded brain sections underwent dewaxing in xylene followed by rehydration through graded ethanol concentrations. The sections were then stained with the Nissl staining solution (G1430, Solarbio; Beijing, China) at 55°C for 20 min. After rinsing with distilled water, differentiation was conducted in 95% ethanol until optimal contrast between Nissl bodies and background was achieved. Subsequently, sections were dehydrated with absolute ethanol, cleared in xylene, and mounted with neutral balsam for microscopic observation. Quantitative analysis of Nissl-positive neurons was performed in three randomly selected fields per section using ImageJ software.

Immunohistochemistry staining was used to measure HMGB1 and caspase1 levels in brain tissues. After pretreatment with antigen retrieval, primary antibodies, caspase1 (1:200; 22915-1-AP, Proteintech, Wuhan, China) and HMGB1 (1:350; ab79823, Abcam, Cambridge, United Kingdom), were incubated on the slices for an entire night at 4°C. Following the incubation of secondary antibodies (PV-9000, ZSGB-BIO, Beijing, China) and the DAB kit (ZLI-9019, ZSGB-BIO, Beijing, China), brain sections were examined and captured under an optical microscope and analyzed using ImageJ analysis.

### 2.6 Ultrastructural assessment of brain tissue using transmission electron microscopy

The ultrastructural alterations in brain tissue were observed using TEM. Tiny tissue samples (1 mm^3^) were extracted from the ischemic penumbra of rat brains. Following an overnight deposition of 2.5% glutaraldehyde at 4°C and 2 h of room-temperature fixation with 1% osmium tetroxide, infiltration with a mixture of graded ethanol and acetone, and then embedding resin, 70-nm slices were coated in lead citrate and uranyl acetate. TEM (HT7700, Japan) was utilized to inspect and photograph sections.

### 2.7 OGD/R model and pharmacological treatments

Rat HAPI microglial cells (YS1284C, YaJi Biological, Shanghai, China) were cultured in a full medium consisting of 10% fetal bovine serum (S711-001S, Lonsera, Shanghai, China), high-glucose DMEM without phenol red (PYG0095, Boster, Wuhan, China), and 1% penicillin-streptomycin solution (P1400, Solarbio, Beijing, China). The incubator was maintained at 37°C and 5% CO_2_ and was humidified. Cells were transferred every 2–3 days, and those exhibiting healthy morphology during the exponential growth phase were chosen for further experiments.

As in the OGD/R model, following a 24-h pretreatment with calycosin, EBSS (CC0041, Leagene, Beijing, China) was added to the solution, and it was then put in a stable-temperature tri-gas incubator (Forma steri-cycle i160 LK, Thermo, United States; 37°C, 1% O_2_, 94% N_2_, and 5% CO_2_). After 5 h in a sugar-free and oxygen-free environment, the medium was replaced with full medium and placed in a regular incubator to mimic reoxygenation. Experiments were performed 24 h after reoxygenation. The control group wasn’t given OGD/R.

To investigate how the NLRP3 inflammasome-mediated pyroptosis pathway contributes to calycosin’s neuroprotective effects on OGD/R damage in microglial cells, the NLRP3-specific inhibitor MCC950 was employed. Cells were separated into five groups: control, OGD/R, calycosin, MCC950, and calycosin + MCC950. MCC950 (10 μM) was added 2 h before OGD.

To further verify calycosin’s efficacy to mitigate LPS and Nigericin-induced pyroptosis, the following groups were included: LPS, LPS + Nigericin, and LPS + Nigericin + calycosin. The cells were deprived of serum for 12 h and subsequently induced with 1 μg/mL LPS for 4 h and 10 μM Nigericin for 1 h before sampling.

### 2.8 CCK8

To assess cell survival, the CCK8 kit (BS350B, Biosharp, Beijing, China) was applied. After seeding the 96-well plates at a density of 50,000/mL, they were pretreated with calycosin and/or OGD/R, added 10 μL of CCK8 solution per well, and subsequently kept at 37°C. After 2 h of incubation at 37°C, the absorbance of the cells was determined at 450 nm using a scanner for microplates (INFINITE 200 PRO, TECAN, Austria). Cell viability (%) was calculated using the following formula:

$$\left[\left(\text{mean of experimental group absorbance}-\text{mean of blank group absorbance}\right)\;/\;\left(\text{mean of control group absorbance}-\text{mean of blank group absorbance}\right)\right]\times 100\%.$$

The control group was set as the reference with 100% viability.

### 2.9 Cell transfection

The small interfering RNAs (siRNAs), including negative control (siNC), fluorescently labeled negative control (siNC-FAM), and three HMGB1-targeting siRNAs (siHMGB1), were purchased from Hippobio (Huzhou, China). The siRNAs sequences are listed in Supplementary Table S1. HAPI cells or HT22 cells (ZQ0476, Shanghai Zhong Qiao Xin Zhou Biotechnology Co., Ltd., Shanghai, China) were seeded in 6-well plates at a density of 2 × 10^5^ cells per well. According to the manufacturer’s protocol, 20 μM stock solutions of siHMGB1, siNC, and siNC-FAM were diluted to a final concentration of 50 nM using Opti-MEM Reduced-Serum Medium (31985070, Gibco, United States). The diluted siRNAs were gently mixed with Lipofectamine 3000 transfection reagent (L3000015, Thermo Fisher Scientific, United States) and incubated at room temperature for 15 min. After adding the transfection mixture to HAPI cells or HT22 cells, the cells were maintained in a standard incubator (37°C, 5% CO_2_) for 12 h, followed by OGD 5h/R 24 h. Transfection efficiency was verified by fluorescence microscopy imaging of siNC-FAM. Both Cells and culture supernatants were harvested for subsequent qRT-PCR, Western blotting, and ELISA to evaluate the extent of HMGB1 knockdown and alterations in pyroptosis-related markers.

### 2.10 Scanning electron microscopy analysis of HAPI cells following OGD/R and calycosin treatment

The ultrastructural morphological alterations in HAPI microglia exposed to calycosin under OGD/R conditions were examined using SEM. HAPI cells were sown on glass coverslips, pretreated with calycosin, and given OGD/R. Following an overnight deposition of 2.5% glutaraldehyde at 4°C and 2 h of room temperature fixation with 1% osmium tetroxide, the samples were dried, dehydrated using a series of grades of ethanol, and covered in a layer of gold film. Finally, the samples were examined and imaged using a SEM (SU8100, Hitachi, Japan) at 3.0 kV, providing high-resolution images of the cell surface and ultrastructure.

### 2.11 Immunofluorescence staining of HAPI cells

Immunofluorescence was chosen to measure HMGB1, NLRP3, and caspase1 levels and identify HAPI microglial cells. Following a 15-min room temperature treatment with 4% paraformaldehyde, 20 min of infiltration with immunostaining permeabilization buffer (BL935B, Biosharp, Beijing, China), and 2 h of room temperature blocking with 5% BSA (SW3015, Solarbio, Beijing, China), primary antibodies such as NLRP3 (1:200; WL02635, Wanlei, Shenyang, China), caspase1 (1:200; 22915-1-AP, Proteintech, Wuhan, China), HMGB1 (1:200; ab79823, Abcam, Cambridge, United Kingdom), and Iba-1 (1:300; 10904-1-AP, Proteintech, Wuhan, China) were added to the samples and incubated for a whole night at 4°C. During 1 h of incubation with secondary antibodies (1:200; A0423, Beyotime Biotechnology, Shanghai, China) and blocking with DAPI-containing solvent to prevent quenching, the slides were examined and captured using an Olympus DP73 fluorescence inverted microscope from Japan and a Zeiss LSM710 laser scanning confocal microscope from Germany. Data were analyzed using ImageJ analysis. Microglial cell purity was determined by counting Iba-1-positive and DAPI-positive cells in at least three random fields, with purity calculated as

$$\left[\text{Iba}-1\left(+\right)/\text{ DAPI}\left(+\right)\right]\times 100\%.$$

### 2.12 ELISA

In accordance with the instructions, the supernatants were collected after centrifuging the cell culture medium for 20 min at 1,500 rpm in order to identify the following factors: HMGB1 (F3649-A, Fankew, Shanghai, China), IL-1β (F2923-A, Fankew, Shanghai, China), and IL-18 (F3070-A, Fankew, Shanghai, China). The absorbance of the cells was determined at 450 nm using a microplate reader. The standard curves were used to calculate the inflammatory mediators’ concentrations.

### 2.13 RNA extraction and qRT-PCR

For animal brain tissue, ischemic penumbra regions were harvested on ice from each group, with corresponding regions taken from the sham-operated control group. Tissue homogenization was performed using a low-temperature tissue homogenizer (LUKYM-1, LUKACEXUYIQI, Guangzhou, China) at 4°C and 40 Hz for 2 min. For cellular samples, cells were collected after 24 h of reoxygenation treatment.

TRIzol (15596026, Invitrogen, New York, United States) was utilized to extract pure total RNA. The detection kit (MR05101; Monad Biotech, Suzhou, China) was chosen to reverse-transcribe RNA into cDNA. Utilizing the dosage ratio and methodology suggested in the manufacturer’s instructions, SYBR Green PCR Mix (MQ10301; Monad Biotech, Suzhou, China) was selected to measure the pyroptosis-related factor levels on a qRT-PCR machine (CFX96, 788BR07571, Bio-Rad, Singapore). β-actin was chosen as an internal reference, and the target mRNA levels were determined with the 2^−ΔΔCT^ technique. The summary of the primer sequences used is provided in Supplementary Table S2.

### 2.14 Western blot analysis

Brain tissue or HAPI cell samples were homogenized using a VCX130 ultrasonic tissue disruptor (SONIC & MATERIALS, United States) set to 30% power with 5-s bursts and 10-s intervals, repeated for 3–5 cycles while kept on ice. The full protein was extracted from the supernatant by rotating the lysates at 12,000 rpm for 20 min. The lysates were prepared using a mixed RIPA lysis buffer (R0100, Solarbio, Beijing, China). The BCA kit (P0010S, Beyotime Biotechnology, Shanghai, China) was chosen to quantify and standardize the protein concentration in each group. Following SDS-PAGE separation, 30 µg of protein were added to PVDF membranes (IPVH00010, Millipore, United States). Following blocking, primary antibodies, including HMGB1 (1:10,000; ab79823, Abcam, Cambridge, United Kingdom), NLRP3 (1:1,500; WL02635, Wanlei, Shenyang, China), caspase1 (1:1,000; 22915-1-AP, Proteintech, Wuhan, China), GSDMD (including N-GSDMD) (1:1,000; P30823, Abmart, Shanghai, China), β-actin (1:1,000; TA-09, ZSGB-BIO, Beijing, China), and IL-18 (1:1,500; P50519-2R2, Abmart, Shanghai, China) were incubated on the membranes for an entire night at 4°C. After washing, the membranes were incubated at room temperature with the corresponding secondary antibodies (AS003 or AS014, ABclonal, Wuhan, China). The ChemiDocTM XRS device (721BR15649, Bio-Rad, United States) captured the signals using an ECL kit (D046, Bridgen, Beijing, China). ImageJ software was employed to examine band intensities, which were then normalized against β-actin to compare protein expression levels across various groups.

### 2.15 Statistical analysis

All data is given as the average ±standard deviation (SD). The SPSS program, version 26, was chosen for the statistical analyses (IBM, Chicago, IL, United States). Normality was assessed using the Shapiro-Wilk test. For non-normally distributed neurological deficit scores (as indicated by Shapiro-Wilk results), the Mann-Whitney U test was applied. Normally distributed parameters were analyzed with one-way ANOVA followed by Dunnett’s *post hoc* test. Charts and graphs were generated with the help of GraphPad Prism 9. Each experiment was performed with a minimum of three replicates. Statistical significance was defined as a p-value of 0.05 or less.

## 3 Results

### 3.1 Calycosin intracerebroventricular administration exerts neuroprotective effects against CIRI in the MCAO rat model

**FIGURE 1:**
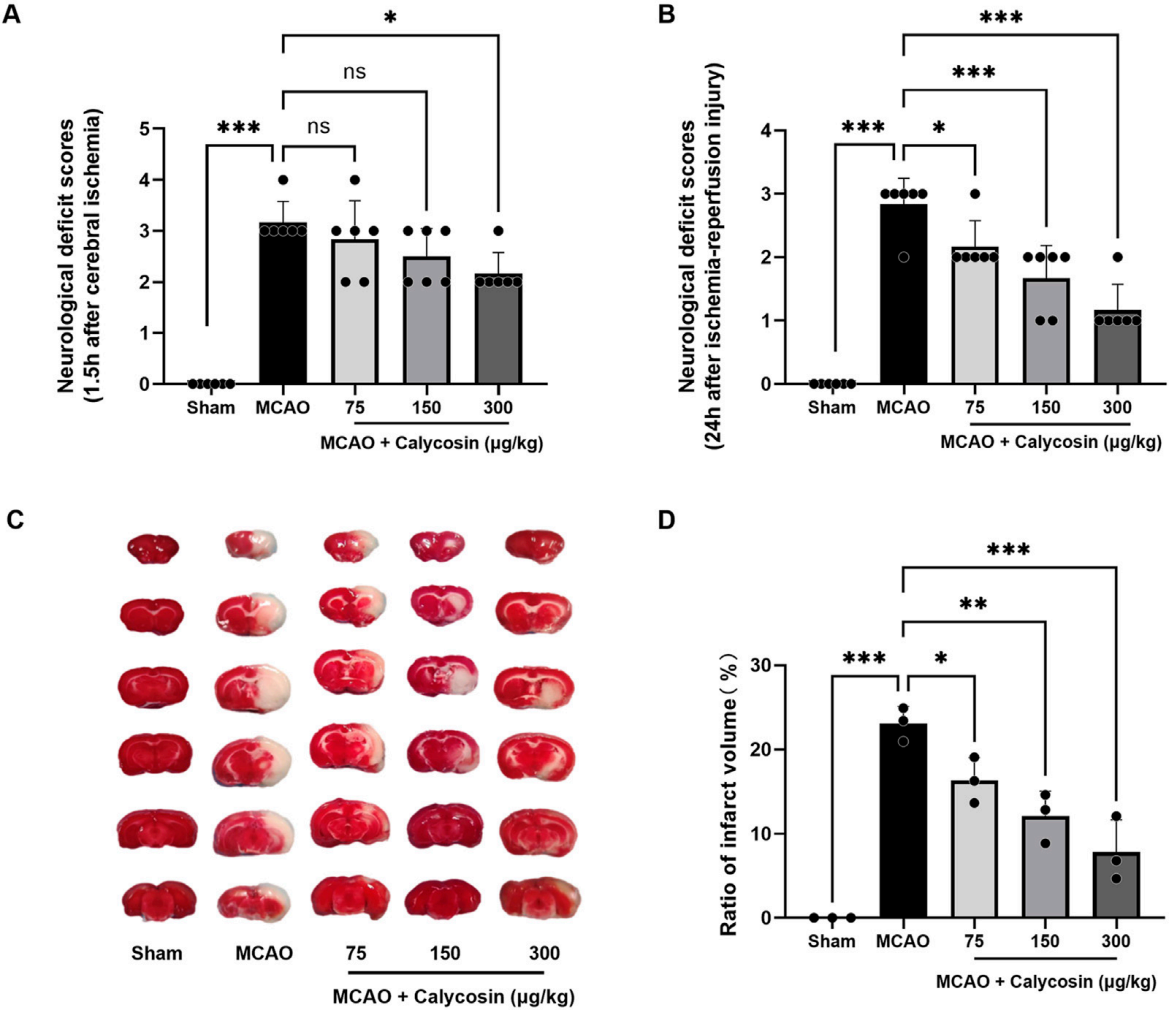
Neuroprotective effects of calycosin intracerebroventricular administration in the MCAO rat model. **(A)** Neurological deficit evaluated using the Zea-Longa scoring method at 1.5 h of cerebral ischemia (n = 6 per group). **(B)** Neurological deficit scores after 1.5 h of cerebral ischemia and 24 h of reperfusion (n = 6 per group). **(C)** Typical brain slices stained with TTC after 1.5 h of cerebral ischemia and 24 h of reperfusion. **(D)** A statistical assessment of the brain slices’ infarct region percent. (n = 3 per group). The mean ± SD is used to express the results. ***p < 0.001, **p < 0.01, and *p < 0.05.

To determine whether calycosin pretreatment could improve neurological function after MCAO-induced injury, a blinded investigator assessed neurological deficits in rats subjected to 1.5 h of ischemia followed by 24 h of reperfusion. Figures 1A,B illustrate that no neurological impairments appeared in the sham-operated group, whereas the MCAO group had markedly higher neurological deficit scores, validating the successful creation of the MCAO model. All calycosin dosages produced neurological deficit scores lower than those of the MCAO group, with the 300 μg/kg dose group showing a significant statistical difference. The pretreatment with calycosin led to a dose-dependent reduction in the scores after 24 h of reperfusion (p < 0.05 or p < 0.001).

TTC staining was applied to brain tissue to determine the degree of the infarction in each group 24 h after reperfusion. The model group had distinct white infarct spots in the brain slices, but the sham group had none at all, as seen in Figure 1C. Following calycosin intracerebroventricular administration, the infarct sizes were reduced to varying degrees. Quantitative analysis (Figure1D) showed that the infarct regions in the model group, compared to the sham group, were significantly larger, reaching 23.09% ± 2.01% (p < 0.001). In the calycosin-treated groups, the proportion of cerebral infarct region decreased in a dose-dependent way as contrasted with the model group, decreasing to 16.33% ± 2.71%, 12.10% ± 2.95%, and 7.84% ± 3.82%, respectively.

These results indicate that calycosin administration intracerebroventricularly provides protection against CIRI in rats.

### 3.2 Calycosin intracerebroventricular administration improves brain tissue injury and ultrastructure damage in the ischemic penumbra region of MCAO model rats

**FIGURE 2:**
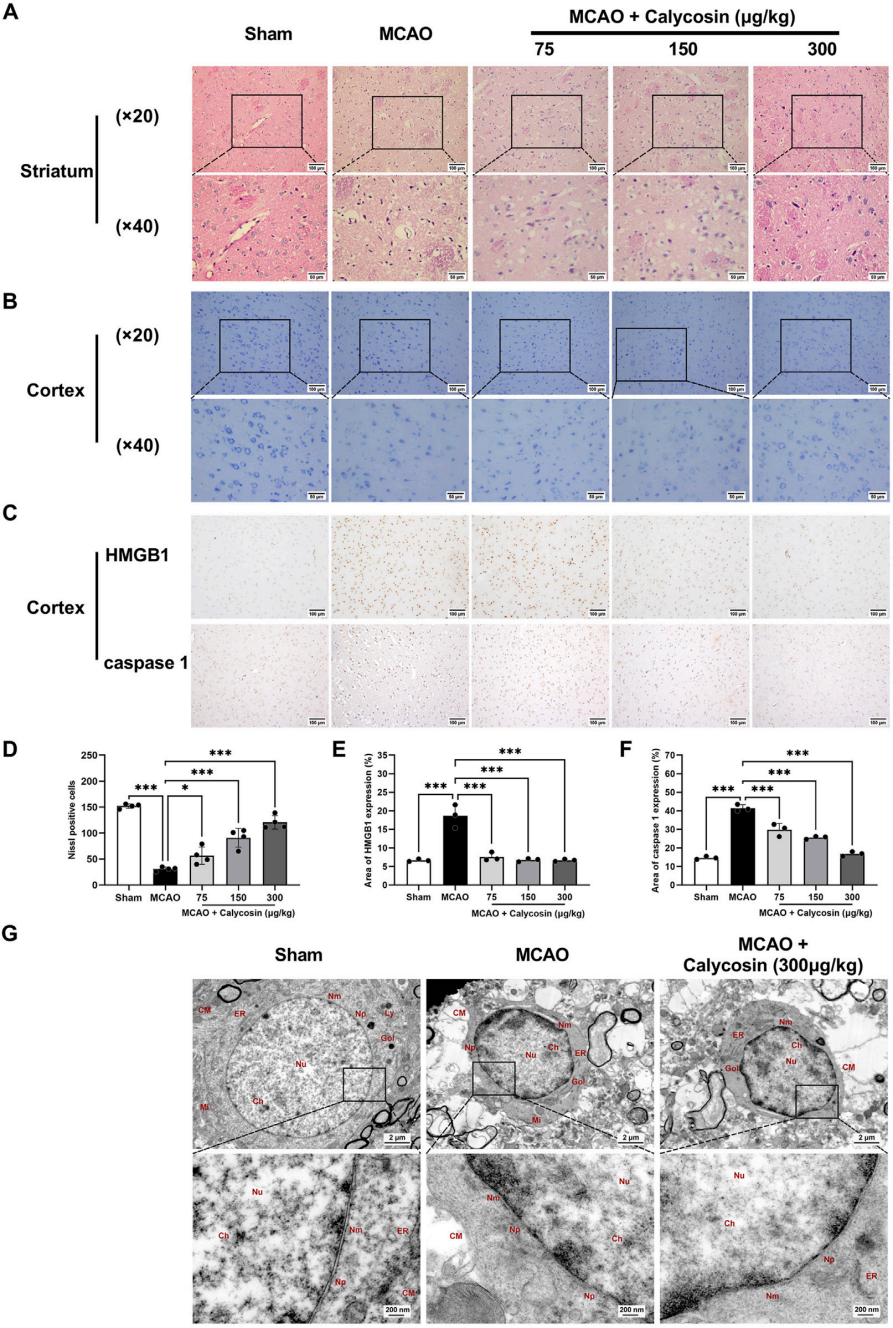
Calycosin intracerebroventricular administration improves morphological damage and ultrastructural changes in the ischemic penumbra and reduces the positive levels of HMGB1 and caspase 1 in brain tissue. **(A)** H-E-stained representative pictures of the striatum region in the ischemic penumbra. Scale bar: (×20) = 100 μm, (×40) = 50 μm. **(B)** Representative Nissl staining images of the cortical region in the ischemic penumbra. Scale bar: (×20) = 100 μm, (×40) = 50 μm. **(C)** Representative images of immunohistochemical staining for HMGB1 and caspase 1 protein expression in brain tissue sections. Scale bar: 100 μm. **(D)** Quantitative analysis of Nissl-positive neurons in the ipsilateral cortex was performed under a 20× light microscope (n = 4 per group). **(E, F)** Semi-quantitative statistical results of immunohistochemical sections of HMGB1 and caspase 1 (n = 3 per group). The mean ± SD is used to express the results. ***p < 0.001, and *p < 0.05. **(G)** Representative images of the cortical area in the ischemic penumbra under a transmission electron microscope. CM: cell membrane, Ch: chromatin, Nu: nucleus, Nm: nuclear membrane, Np: nuclear pore, Mi: mitochondria, Gol: Golgi apparatus, Ly: lysosome, ER: endoplasmic reticulum. Scale bar: 2 μm (upper panel), 200 nm (lower panel).

H-E staining was utilized to examine tissue alterations in the striatum within the ischemic penumbra (Figure 2A). In the sham group, neurons were orderly arranged with intact morphological structures. In the MCAO group, numerous degenerative and necrotic neurons were observed, with a significant reduction in cell number, severe vacuolar degeneration, and nucleolar shrinkage. In the calycosin-treated groups, the number of neurons rose, while the level of swelling and necrosis was ameliorated in the ischemic penumbra region.

Ischemic brain injury in rats was assessed by Nissl staining of the cortex ischemic penumbra (Figures 2B,D). The sham group exhibited normal neuronal morphology with distinct cytoplasmic and nuclear boundaries, and orderly arranged Nissl bodies. In the MCAO group, the neuronal cells showed loose arrangement, abnormal morphology, a reduced cell density, a decreased Nissl body counts, and partial Nissl body dissolution. Calycosin pretreatment dose-dependently ameliorated these pathological alterations, including recovery of Nissl body counts, neuronal morphology, and cell density.

Immunohistochemical analysis of the cerebral cortex ischemic penumbra (Figures 2C,E,F) revealed that the MCAO group had a considerably higher proportion of HMGB1 and caspase1-positive cells than the sham group. In contrast, calycosin pretreatment resulted in a dose-dependent reduction in the proportion of HMGB1 and caspase1-positive cells compared to the MCAO group. Calycosin pretreatment reduced the number of HMGB1 and caspase1-positive cells in a dose-dependent manner compared to the MCAO group.

Using transmission electron microscopy, ultrastructural alterations in neurons inside the cortical ischemia penumbra were investigated (Figure 2G). Neurons in the sham group exhibited normal morphology, with intact cell membranes and no swelling or destruction of intracellular organelles. In the MCAO group, cells were swollen and deformed, with severe membrane damage and numerous membrane pores, and organelles were swollen and dissolved. Following calycosin pretreatment, the extent of neuronal damage was reduced, with relatively intact cell membranes and improved organelle integrity. Based on rats that had cerebral ischemia-reperfusion injuries, this shows that calycosin may have neuroprotective effects by inhibiting neuroinflammation and pyroptosis.

### 3.3 Calycosin intracerebroventricular administration alleviates CIRI in rats by suppressing HMGB1/NLRP3-mediated pyroptosis

To further investigate whether the neuroprotective effects of intracerebroventricular injection of calycosin against CIRI in rats are associated with the HMGB1/NLRP3-mediated pyroptosis signaling pathway, we extracted total RNA and protein from the ischemic penumbra of brain tissues from each group of rats at 24 h reperfusion following 1.5 h of cerebral ischemia and conducted qRT-PCR and Western blot analyses.

**FIGURE 3:**
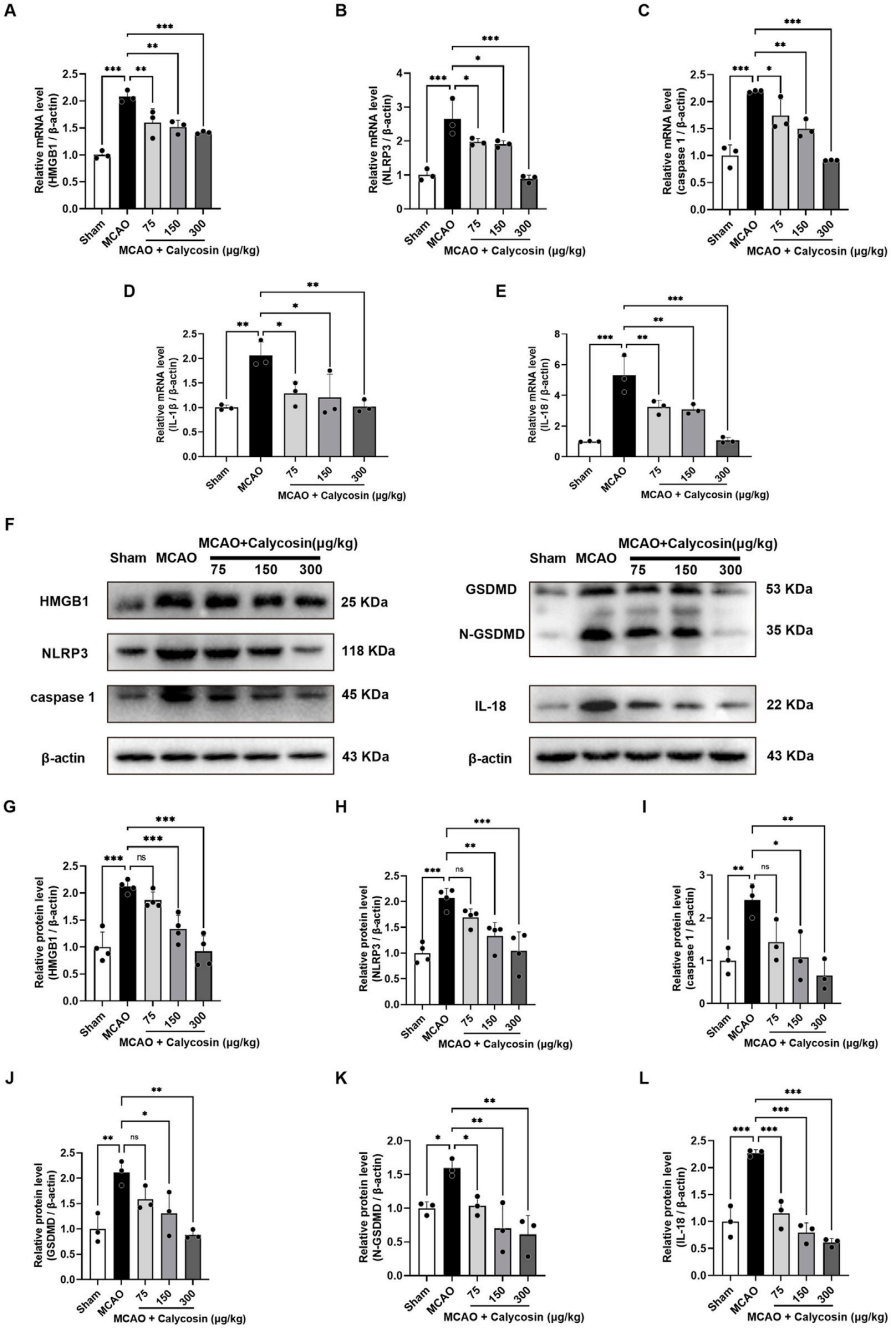
Calycosin intracerebroventricular administration reduces the mRNA and protein expression of molecules related to cell pyroptosis in the brain tissue of rats in the MCAO model. **(A–E)** Effects of calycosin on HMGB1, NLRP3, caspase1, IL-1β, and IL-18 mRNA expression levels in rats with CIRI. β-actin was used as an internal reference. **(F–L)** Effects of calycosin on the levels of NLRP3, caspase1, HMGB1, GSDMD, N-GSDMD, and IL-18 protein expression in the CIRI rats. β-actin served as the internal standard. The mean ± SD is used to express the results. ***p < 0.001, **p < 0.01, and *p < 0.05. n = 3-4 per group.

As illustrated in Figures 3A–E, the mRNA expression levels of HMGB1, NLRP3, caspase1, IL-1β, and IL-18 were significantly greater in the MCAO group compared to the sham group. A considerable rise was seen in the model group as compared to the sham group based on the quantitative assessment of HMGB1, NLRP3, caspase1, GSDMD, N-GSDMD, and IL-18 bands (Figures 3F–L). Notably, calycosin intervention resulted in a dose-dependent decrease in the mRNA and protein expression of these pyroptosis-associated markers.

The results indicate that calycosin intracerebroventricular administration could offer neuroprotection against CIRI in rats by inhibiting the HMGB1/NLRP3-driven pyroptosis signaling pathway.

### 3.4 Calycosin enhances cell viability and mitigates pyroptotic damage in HAPI cells under OGD/R conditions

**FIGURE 4:**
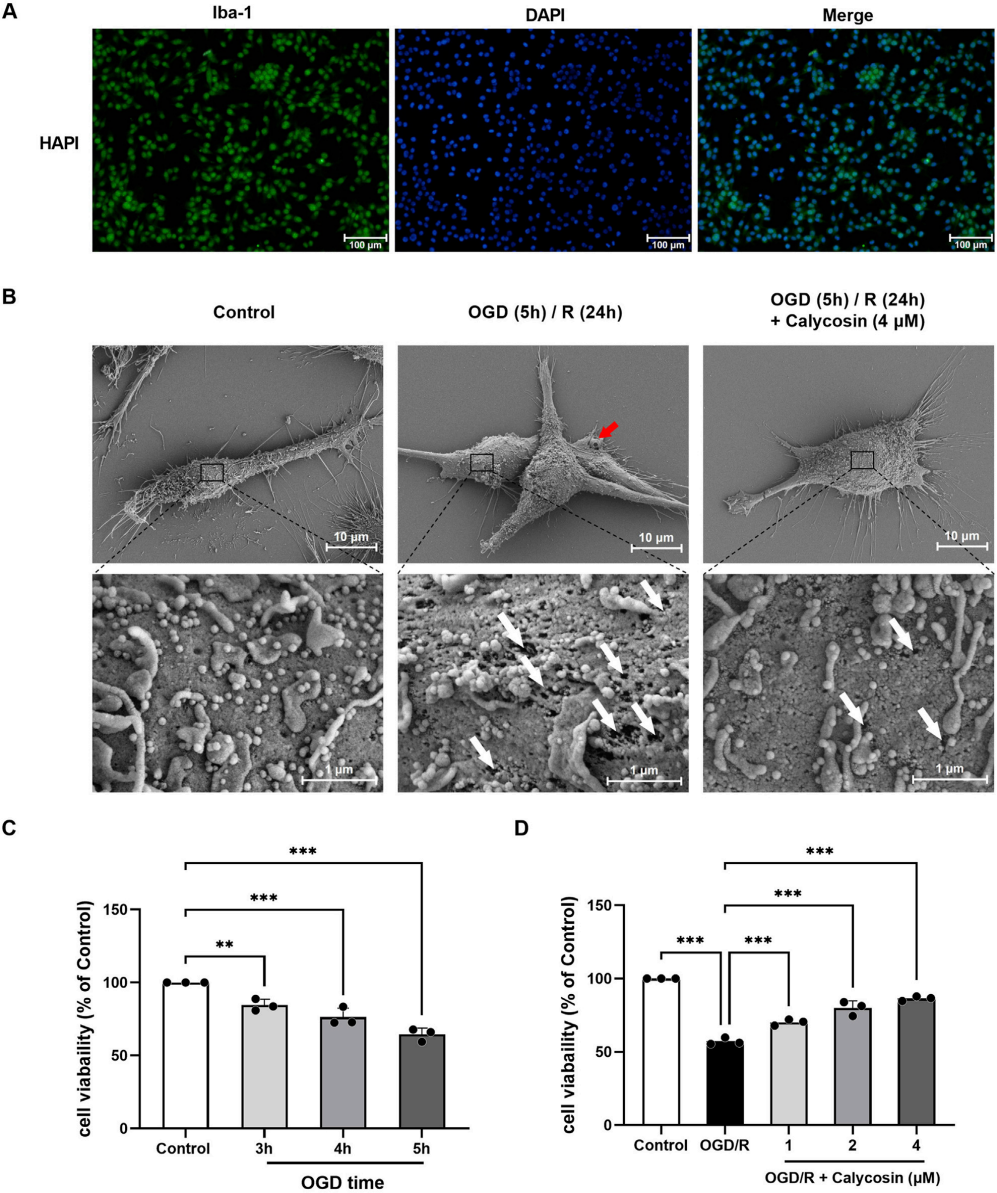
Identification of HAPI cells, observation of cellular ultrastructure via scanning electron microscopy, and optimization of OGD duration and calycosin concentration. **(A)** Identification of HAPI microglia. DAPI (blue), Iba-1 (green). 100 μm is the scale bar. **(B)** Ultrastructure of HAPI cells observed under scanning electron microscopy in the control group, OGD (5 h)/R (24 h) model group, and 4 μM calycosin pre-treatment group. The red arrow indicates the pyroptotic bubbles, and the white arrow indicates the pyroptotic membrane pore. Upper panel scale bar: 10 μm; lower panel scale bar: 1 μm. **(C)** Effect of different OGD durations on the viability of HAPI microglia, assessed by the CCK8 assay. **(D)** Effect of various calycosin concentrations on the viability of HAPI microglia following OGD (5 h)/R (24 h), assessed by the CCK8 assay. The mean ± SD is used to express the results. ***p < 0.001, and **p < 0.01. n = 3 per group.

To confirm if calycosin’s neuroprotective properties are linked to pyroptosis, we utilized rat HAPI microglia exposed to OGD/R as an *in vitro* CIRI model. Log-phase HAPI cells were cultured and assessed for purity using immunofluorescence staining with the microglial-specific antibody Iba-1. The results indicated a cell purity of over 95%, making them suitable for subsequent experiments (Figure 4A).

Using scanning electron microscopy, it was observed that the HAPI cell line in the OGD/R group, contrasting to the control group, showed notable swelling, extensive membrane damage, bubble-like protrusions (pyroptotic bodies marked by red arrows), and many irregular membrane pores (white arrows) after 5 h of OGD and 24 h of reoxygenation and glucose restoration, which are characteristic of pyroptotic morphology. The calycosin pre-treatment group (4 μM) showed a significant reduction in membrane pores and pyroptotic bodies, indicating that calycosin alleviates OGD/R-induced pyroptosis in microglial cells (Figure 4B).

Exposure of HAPI cells to OGD for durations of 3, 4, and 5 h led to a progressive reduction in cell viability, depending on the CCK8 kit, with viability dropping to 84.53%, 76.26%, and 64.44%, respectively (Figure 4C). Based on the observed moderate cell damage and experimental conditions, we selected 5 h of OGD and 24 h of reoxygenation and glucose restoration for subsequent modeling.

Under OGD (5 h)/R (24 h) conditions, the CCK8 test was employed to evaluate the HAPI cells’ viability at varying drug dosages (1–4 μM). The results showed that calycosin provided neuroprotection in the OGD/R model, with its effectiveness varying according to the dosage (Figure 4D).

### 3.5 Calycosin inhibits the HMGB1/NLRP3-mediated pyroptosis pathway and the triggered inflammatory response in OGD/R-induced HAPI microglial damage

**FIGURE 5:**
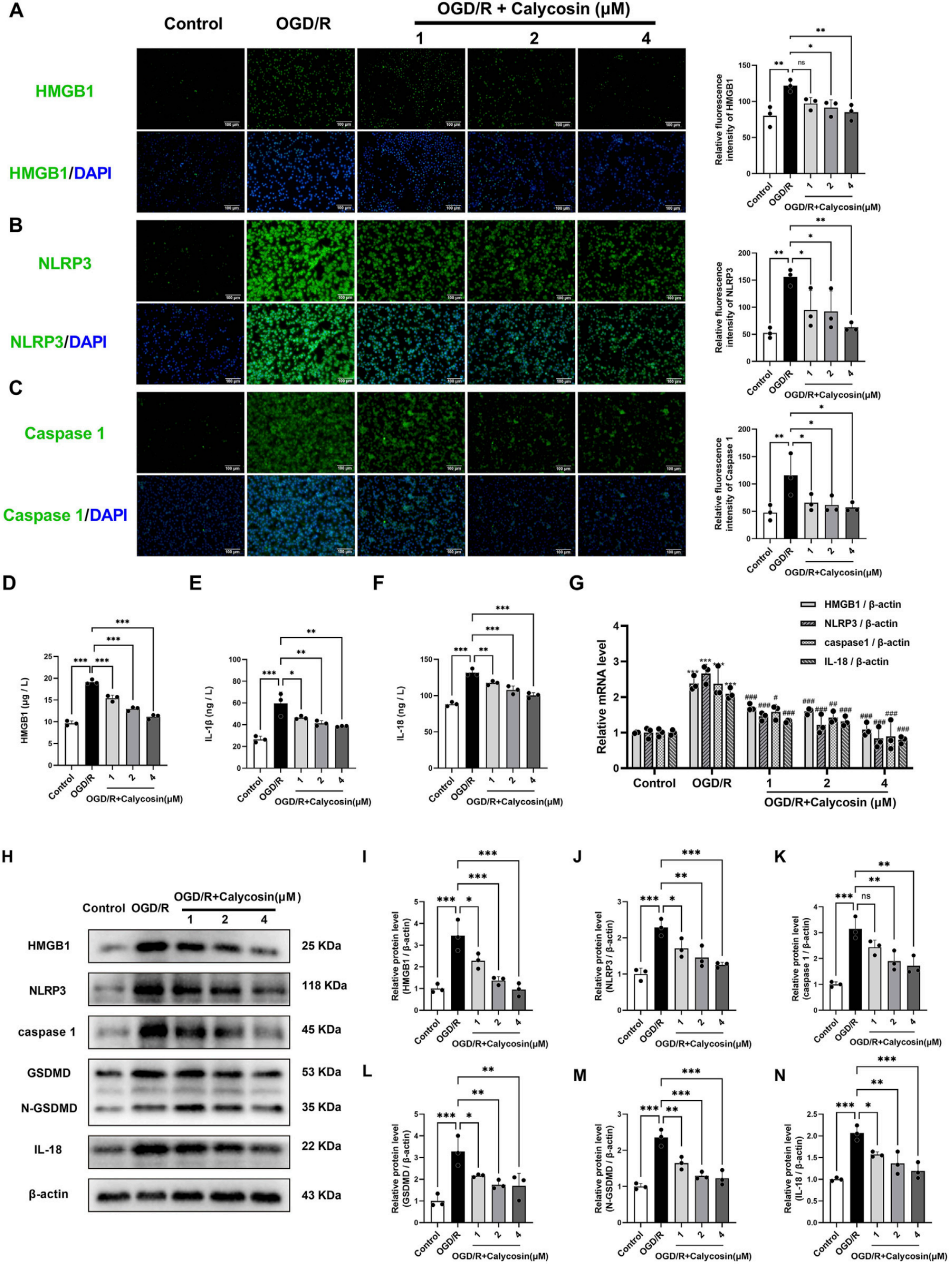
Calycosin protects against CIRI by blocking the HMGB1/NLRP3-mediated pyroptosis pathway and the pyroptosis-related inflammatory cascades in OGD/R-treated HAPI cells **(A–C)** Representative immunofluorescence images and fluorescence intensity analysis of HMGB1 (green), NLRP3 (green), caspase-1 (green), and DAPI (blue) in HAPI microglia across control, OGD/R, and OGD/R + calycosin (1, 2, 4 μM) pretreatment groups. Scale bar = 100 μm. **(D–F)** ELISA measurements of inflammatory cytokines (HMGB1, IL-18, and IL-1β) levels in HAPI microglia supernatants from each group. ***p < 0.001, **p < 0.01, and *p < 0.05. n = 3 per group. **(G)** The effect of calycosin on HMGB1, NLRP3, caspase1, and IL-18 mRNA expression levels in HAPI microglia following OGD/R injuries. A β-actin internal control was employed. ***p < 0.001, the OGD/R group vs. the control group; ^###^p < 0.001, ^##^p < 0.01, and ^#^p < 0.05, the calycosin treatment groups vs. the OGD/R group. n = 3 per group. **(H–N)** The effect of calycosin on HMGB1, NLRP3, caspase1, GSDMD, N-GSDMD, and IL-18 protein expression levels in HAPI microglia following OGD/R injuries. A β-actin internal control was employed. ***p < 0.001, **p < 0.01, and *p < 0.05. n = 3 per group. The mean ± SD is used to express the results.

We conducted immunofluorescence staining to examine if calycosin’s protective impact on HAPI microglia is linked to the modulation of pyroptosis-related inflammatory pathways by measuring the expression levels of HMGB1, NLRP3, and caspase1. When compared to the control group, the OGD/R group’s fluorescence levels for these compounds showed a substantial increase. However, calycosin pretreatment markedly reduced immunofluorescence levels of HMGB1, NLRP3, and caspase1 (Figures 5A–C). To further characterize the spatial dynamics of these pyroptosis-related proteins, laser scanning confocal microscopy was employed to examine the subcellular localization of HMGB1, NLRP3, and caspase-1 in HAPI cells (Supplementary Figure S1). High-resolution imaging revealed predominant nuclear localization of HMGB1 in untreated control cells. OGD/R exposure significantly enhanced HMGB1 expression and triggered its nuclear-to-cytoplasmic translocation, which was markedly attenuated by 4 μM calycosin administration. Both NLRP3 and caspase-1 exhibited cytoplasmic localization, with their expression levels being significantly upregulated following OGD/R and effectively downregulated by calycosin (4 μM). A negative control lacking primary antibodies demonstrated exclusive DAPI-stained nuclei (blue fluorescence), confirming the specificity of immunostaining results.

We used an ELISA kit to detect the amounts of the inflammatory mediators HMGB1, IL-1β, and IL-18. Figures 5D–F demonstrate that, compared to the control group, OGD/R significantly increased three inflammatory mediators in supernatants: HMGB1 levels rose 2.0-fold to 19.1 μg/L, IL-1β increased 2.2-fold to 59.6 ng/L, and IL-18 showed a 1.5-fold elevation reaching 131.6 ng/L. Calycosin pre-treatment produced dose-dependent reductions in these inflammatory mediators versus the OGD/R group. At 1, 2, and 4 μM doses, HMGB1 levels decreased to 80.6%, 67.8%, and 58.3% of OGD/R values; IL-1β to 78.0%, 69.3%, and 64.6%; and IL-18 to –89.2%, 81.8%, and 76.1%, respectively. These findings imply that calycosin may shield microglia following OGD/R by preventing inflammatory cascades linked to pyroptosis.

To determine if calycosin pre-treatment shields HAPI cells from OGD/R injury through the HMGB1/NLRP3 pyroptosis pathway, we gathered cell samples to extract total RNA and protein, then performed qRT-PCR and Western blot tests. Figure 5G illustrates that the OGD/R group’s HMGB1, NLRP3, caspase1, IL-1β, and IL-18 mRNA expression levels were significantly higher than those of the control group. However, the pyroptosis-related molecules listed above decreased in a dose-dependent manner following calycosin intervention.

Likewise, as illustrated in Figures 5H–N, densitometric analysis of HMGB1, NLRP3, caspase1, GSDMD, N-GSDMD, and IL-18 bands, compared to the control group, revealed an appreciable rise in the OGD/R group. Notably, the protein expression of these pyroptosis-related molecules was dose-dependently decreased following calycosin intervention.

The results indicate that calycosin might offer protection against brain ischemia-reperfusion damage by blocking the HMGB1/NLRP3-driven pyroptosis mechanism in HAPI microglial cells.

### 3.6 Calycosin alleviates OGD/R-induced pyroptosis in HAPI microglia by blocking HMGB1-dependent NLRP3 inflammasome activation

We investigated if calycosin could reduce OGD/R-triggered pyroptosis in HAPI cells by blocking NLRP3 inflammasome activation, using the NLRP3 inhibitor MCC950, and evaluating the levels of proteins associated with pyroptosis driven by NLRP3. Based on preliminary experiments and manufacturer recommendations, we pretreated HAPI cells with 4 μM calycosin for 24 h and 10 μM MCC950 for 2 h.

**FIGURE 6:**
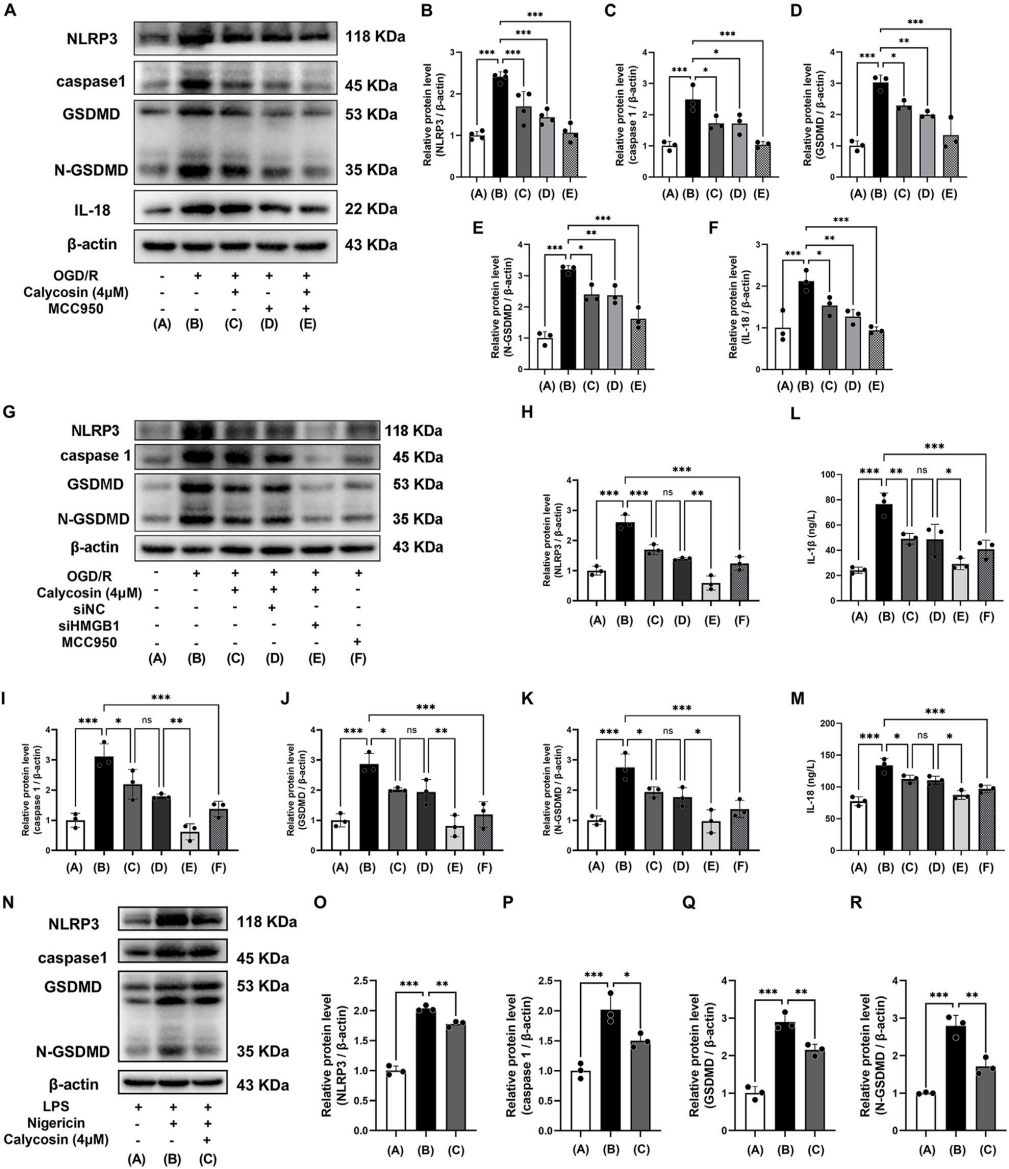
Calycosin ameliorates HAPI cell pyroptosis induced by OGD/R or LPS/Nigericin through inhibition of NLRP3 inflammasome activation. **(A–F)** Western blot analysis was used to assess how calycosin, MCC950, and their combination affected the amounts of NLRP3, caspase1, GSDMD, N-GSDMD, and IL-18 protein expression in HAPI microglia following OGD/R. **(G–M)** Calycosin effects on the pyroptosis marker proteins (NLRP3, caspase1, GSDMD, and N-GSDMD) and the inflammatory cytokines (IL-1β and IL-18) release in siHMGB1-transfected HAPI microglia following OGD/R. **(N–R)** Western blot analysis was used to assess the protein expression of NLRP3, caspase1, GSDMD, and N-GSDMD in HAPI microglia following NLRP3 inflammasome activation induced by LPS/Nigericin in the presence or absence of 4 μM calycosin. A β-actin internal control was employed. The mean ± SD is used to express the results. ***p < 0.001, **p < 0.01, and *p < 0.05. n = 3-4 per group.

Western blot analysis demonstrated that calycosin or MCC950 pretreatment significantly reduced protein levels of NLRP3, caspase 1, GSDMD, N-GSDMD, and IL-18 in comparison to the OGD/R group, with equivalent inhibitory efficacy between the two treatments. Both calycosin and MCC950 efficiently suppressed the expression of downstream proteins in the NLRP3-mediated pyroptosis pathway when administered together. Moreover, the combined treatment enhanced the inhibitory effect on NLRP3 inflammasome activation (Figures 6A–F).

Given the well-established role of HMGB1 in regulating NLRP3 inflammasome activity, we further explored whether calycosin’s protective effects were mediated through HMGB1 signaling. To address this, HMGB1 expression was specifically knocked down in HAPI cells using siRNA, enabling systematic evaluation of OGD/R-induced pyroptosis markers and calycosin’s pharmacology under HMGB1-deficient conditions. The Western blot results demonstrated that OGD/R significantly upregulated HMGB1 downstream effectors (NLRP3, caspase-1, GSDMD and N-GSDMD), whereas HMGB1 siRNA pretreatment substantially attenuated these pyroptosis-related protein expressions (Figures 6G–K). ELISA quantification further validated that HMGB1 silencing significantly diminished IL-1β and IL-18 release, demonstrating HMGB1’s pivotal role in OGD/R-induced pyroptosis (Figures 6L,M). Notably, calycosin (4 μM) treatment alone attenuated pyroptosis markers and inflammatory cytokine release. The combination of calycosin and HMGB1 siRNA showed synergistic suppression, indicating that calycosin’s neuroprotection partially depends on HMGB1-NLRP3 axis modulation. The HMGB1 knockdown experiments in HT22 cells yielded consistent results with those in HAPI cells (Supplementary Figures S2, S3).

To confirm if calycosin can mitigate NLRP3 inflammasome activation triggered by LPS and nigericin, we performed additional tests. We pre-treated the cells with 4 μM calycosin for 24 h and subsequently treated the cells with 10 μM nigericin for 1 h after 4 h of 1 μg/mL LPS exposure, based on initial experiments and manufacturer instructions.

Western blot examination revealed that the LPS/Nigericin-treated group had significantly higher levels of NLRP3, caspase1, GSDMD, and N-GSDMD proteins than the LPS control group, indicating activation of the NLRP3-driven pyroptosis pathway. Following calycosin (4 μM) intervention, the levels of these pyroptosis-related proteins dramatically decreased, suggesting that LPS and nigericin-induced NLRP3 inflammasome activation can be reversed by calycosin (Figures 6N–R).

The results indicate that calycosin’s neuroprotective effects are mediated through HMGB1-dependent NLRP3 inflammasome suppression, thereby alleviating microglial pyroptosis.

## 4 Discussion

**FIGURE 7:**
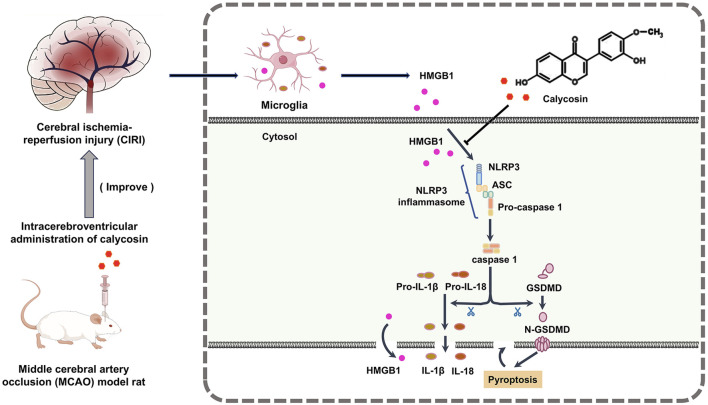
Mechanism diagram of the protective effect of calycosin against the HMGB1-dependent pyroptosis pathway in rats with CIRI.

This research investigated the neuroprotective properties and possible mechanisms of calycosin, an isoflavone phytoestrogen from Astragalus, in a rat model of CIRI caused by MCAO. The results indicated that intracerebroventricular injection of calycosin dramatically improved the state of neurologic deficits about neurological impairments and infarct size in the MCAO model. Moreover, calycosin reduced both the structural and ultrastructural damage in the ischemic penumbra and inhibited the levels of HMGB1, the NLRP3 inflammasome, and its downstream effectors such as caspase1, GSDMD, IL-1β, and IL-18 in the cerebral ischemic penumbra of MCAO rats. Laboratory experiments further demonstrated that calycosin improved the survival rate of HAPI microglial cells following OGD/R, inhibited cell pyroptosis, and decreased expression of HMGB1, NLRP3, caspase1, and IL-1β and IL-18 levels. These results indicate that calycosin exerts neuroprotective effects by suppressing HMGB1-NLRP3-mediated pyroptosis in CIRI (The mechanism diagram is shown in Figure 7).

Much interest has been focused on the function of HMGB1-NLRP3-mediated pyroptosis in the pathophysiology of ischemia-reperfusion damage (Ismael et al., 2021; Zhu et al., 2021b; Ding et al., 2023). HMGB1, an essential extracellular DAMP signaling protein, is mainly driven by the assembly of the NLRP3 inflammasome, a complex of proteins that activates caspase-1, finally causing IL-1β and IL-18 to mature and secrete. This process promotes pyroptotic cell death, which exacerbates brain tissue damage (Liu et al., 2022; Liao et al., 2024). In line with our results, earlier research has shown that blocking NLRP3 inflammasome-driven pyroptosis can significantly reduce brain ischemia-reperfusion injury. According to Franke et al. (2021), in a mouse model suffering from CIRI brought on by transient middle cerebral artery blockage (tMCAO), there was a notable increase in both gene and protein expression levels of NLRP3. They found that the NLRP3 inflammasome inhibitor MCC950 significantly reduced cerebral infarct volume, inhibited caspase-1 activation, attenuated inflammation, and stabilized the blood-brain barrier, thereby preventing I/R injury. Similarly, the Yang F et al. study (Yang et al., 2014) showed that lacking NLRP3 improves neurovascular injury in mice with ischemic stroke by lowering IL-1β secretion and oxidative stress driven by NOX2. Moreover, the NLRP3 inflammasome plays a critical role in the ischemia-induced breakdown of the blood-brain barrier. It activates inflammatory cascades, induces pyroptosis of brain endothelial cells, and promotes blood-brain barrier disruption, thus aggravating CIRI. Preventing the early activation or blocking of NLRP3 can mitigate CIRI by decreasing inflammation and strengthening the blood-brain barrier (Bellut et al., 2021).

One of the primary mechanisms of CIRI is the activation of the NLRP3 inflammasome (Barrington et al., 2017; Long et al., 2023). For the prevention and treatment of ischemic stroke, it is imperative to investigate the NLRP3 inflammasome further as a potential therapeutic target (Coll et al., 2022; Yuan et al., 2024). Recent research has discovered that the active ingredients in some traditional Chinese medicine extracts can protect against brain ischemia-reperfusion injury by preventing NLRP3 inflammasome-driven pyroptosis. For instance, Liao et al. (2024) reported that acteoside, a bioactive substance derived from *Cistanche tubulosa*, can significantly reduce inflammation and cell pyroptosis caused by blood-brain barrier damage in CIRI rats. This is achieved by blocking the HMGB1/TLR4/NLRP3 pathway and encouraging microglia to adopt the M2 phenotype. Similarly, the Ma DC et al. study (Ma et al., 2021a) showed that pre-administration of salvianolic acids enhances neuron survival by altering microglial polarization from M1 to M2, thus suppressing the NLRP3 inflammasome/pyroptosis pathway in both laboratory and live models of brain ischemia-reperfusion injury. Our observations align with these results, indicating that calycosin can provide neuroprotection by blocking the HMGB1-NLRP3-driven pyroptosis pathway.

Neuroinflammation is a crucial mechanism in cerebral ischemia-reperfusion injury (Long et al., 2023). Microglia, which constitute 5% of all glial cells, are the intrinsic immune effector cells in the central nervous system and play a significant role in neuroinflammation following ischemic stroke (Barrington et al., 2017). In this study, we found that intracerebroventricular injection of calycosin reduced the expression of inflammatory mediators such as IL-1β and IL-18 in the brain tissue of MCAO rats. Additionally, in the OGD/R *in vitro* model, calycosin improved microglial morphology, alleviated pyroptotic features, and decreased the release of inflammatory mediators. Therefore, calycosin may exert neuroprotective effects by inhibiting excessive microglial activation.

The selective distribution of calycosin in the brain is one of its benefits for its therapeutic potential anti-CIRI activity (Du et al., 2022). Zheng et al. (2020) employed HPLC-MS/MS to analyze the pharmacokinetics of calycosin in both the blood and brain tissues of healthy rats and those with CIRI. The study revealed a greater concentration of calycosin in brain tissue, suggesting its potential for brain-targeted therapy, which is essential for its future clinical use and drug development. Compared to systemic administration, the intracerebroventricular route of administration provides better targeting, avoiding the influence of hepatic and renal metabolism as well as the blood-brain barrier, leading to higher brain tissue concentrations and improved therapeutic efficacy, and providing direct experimental evidence for elucidating the neuroprotective mechanisms of calycosin (Sadekar et al., 2022; Jin et al., 2024). However, intracerebroventricular administration may not be clinically feasible due to its invasiveness and surgical risks. Emerging non-invasive alternatives such as intranasal delivery or nanoparticle systems demonstrate enhanced brain targeting while improving safety and patient compliance (Ronaldson et al., 2024; Du et al., 2025). These approaches could overcome existing limitations in calycosin delivery for ischemic stroke treatment and other neurological disorders.

Accumulating evidence underscores that calycosin protects against CIRI through multiple mechanisms, including anti-apoptotic, ferroptosis-suppressive, and antioxidant activities. Guo et al. demonstrated that calycosin ameliorates CIRI through antioxidant mechanisms, specifically by reducing reactive oxygen species (ROS), suppressing 4-Hydroxy-2-nonenal (4-HNE) expression, and enhancing antioxidant enzyme activity (Guo et al., 2012). Liu et al. further revealed that calycosin binds to ACSL4, thereby inhibiting ferroptosis and attenuating injury (Liu et al., 2023). Our previous research showed that calycosin upregulates the expression of p62, NBR1, and Bcl-2 expression while downregulating TNF-α, suggesting its neuroprotective effects may be mediated through anti-autophagic, anti-apoptotic, and anti-inflammatory (Wang et al., 2018).

Our current study demonstrates that calycosin markedly decreased the levels of HMGB1, NLRP3 inflammasome, and associated downstream molecules in the ischemic penumbra of MCAO rats, aligning with the observed improvements in neurological deficits and smaller infarct areas. Additionally, we directly observed the morphological evidence of pyroptosis, such as membrane pore formation and cell swelling in the CIRI model, which was significantly alleviated by calycosin treatment. These findings suggest that the HMGB1-NLRP3-mediated pyroptotic pathway may be an important target through which calycosin exerts its neuroprotective effects. HMGB1 functions as an upstream activator driving NLRP3 inflammasome assembly, which achieves its extracellular release during pyroptosis to perpetuate the inflammatory cascade. Recent research has shown that calycosin directly binds to HMGB1, altering its secondary structure and environment of the chromogenic amino acids to suppress pro-inflammatory activity (Ye et al., 2023). In this study, HMGB1 siRNA transfection in HAPI and HT22 cells significantly attenuated pyroptosis-related markers (NLRP3, caspase-1, GSDMD, and N-GSDMD) under OGD/R exposure. Notably, the combination of calycosin with HMGB1 siRNA produced synergistic suppression exceeding the effects of HMGB1 siRNA monotherapy. These concordant findings not only establish the mechanistic specificity of pyroptosis inhibition but also suggest that HMGB1 serves as a master regulator in pyroptotic signaling, with calycosin potentially enhancing therapeutic efficacy through complementary pathways. However, further investigation is required to elucidate the distribution of HMGB1 in plasma, cerebrospinal fluid, and cellular compartments during CIRI, and to ascertain whether calycosin inhibits HMGB1 translocation, secretion, or receptor engagement. Notably, our research revealed that calycosin’s NLRP3 inflammasome inhibitory efficacy parallels that of MCC950, an established selective NLRP3 inhibitor. Though both suppressed core pyroptosis markers (NLRP3, caspase-1, GSDMD, N-GSDMD, and IL-18), their combination further attenuated OGD/R-induced NLRP3 activation and exhibited synergistic efficacy. This implies that calycosin’s flavonoid structure may confer additional neuroprotection through alternative mechanisms, particularly via its antioxidant properties. Given ROS are known upstream activators of the NLRP3 inflammasome (Dominic et al., 2022), a plausible mechanism may involve calycosin-mediated ROS scavenging; however, this hypothesis requires experimental verification.

Key limitations must be addressed for clinical translation. Although this study shows calycosin protects microglia via anti-pyroptotic mechanisms, its effects on neurons and astrocytes, as well as crosstalk between pyroptosis and oxidative stress/apoptosis pathways, remain unclear. Current rodent studies focus more on prevention than treatment, with insufficient data on blood-brain barrier permeability. To optimize efficacy and minimize side effects, we must align dosing windows with central nervous system bioavailability through synchronized therapeutic regimens. Future work should emphasize cell-type-specific omics profiling and pharmacokinetic-pharmacodynamic modeling. Successfully addressing these challenges requires tighter integration between mechanistic investigations and clinically viable delivery strategies.

Future studies should prioritize three key approaches to further elucidate calycosin’s neuroprotective mechanisms. Employing HMGB1 or NLRP3 knockout mice models would validate its anti-pyroptotic action. Concurrent pharmacokinetic analyses should quantify cerebral drug levels after systemic administration, particularly focusing on blood-brain barrier penetration dynamics. These analyses should integrate with targeted delivery strategies to enhance therapeutic precision. Parallel single-cell omics could map cell-type-specific responses across neural populations, whereas multi-omics integration may unravel pyroptosis-apoptosis crosstalk. These coordinated efforts will accelerate translation from mechanistic insights to clinically translatable therapies.

## 5 Conclusion

In summary, this study elucidates a novel mechanism by which calycosin exerts neuroprotective effects, likely linked to modulating the HMGB1-NLRP3-mediated pyroptosis pathway by inhibiting excessive microglial polarization. These findings provide vital experimental data for future research into calycosin’s therapeutic potential in ischemic stroke treatment. Future research should focus on optimizing the administration route, expanding the investigation of its mechanisms of action, and conducting more comprehensive animal model validation to lay the groundwork for the clinical translation of calycosin.

## Data Availability

The original contributions presented in the study are included in the article/Supplementary Material, further inquiries can be directed to the corresponding authors.
