# Supplementary figures and images for: Intracerebroventricular calycosin attenuates cerebral ischemia-reperfusion injury in rats via HMGB1-dependent pyroptosis inhibition
Source: Front Pharmacol. 2025 Jun 18;16:1596087. doi: 10.3389/fphar.2025.1596087 (PMC12213575; doi:10.3389/fphar.2025.1596087)

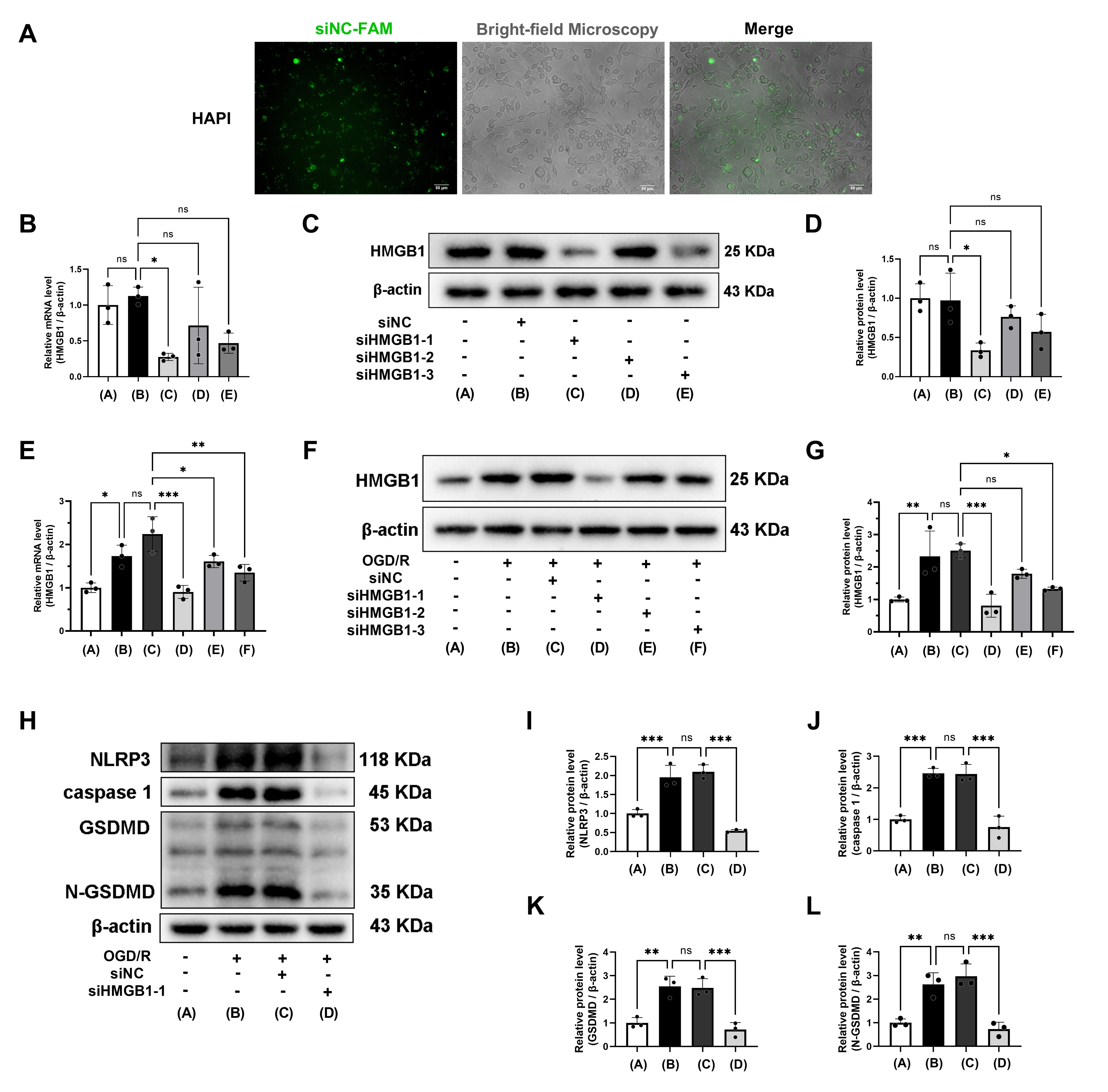

Supplement: Supplementary file 1 [file Supplementaryfile2.jpeg]

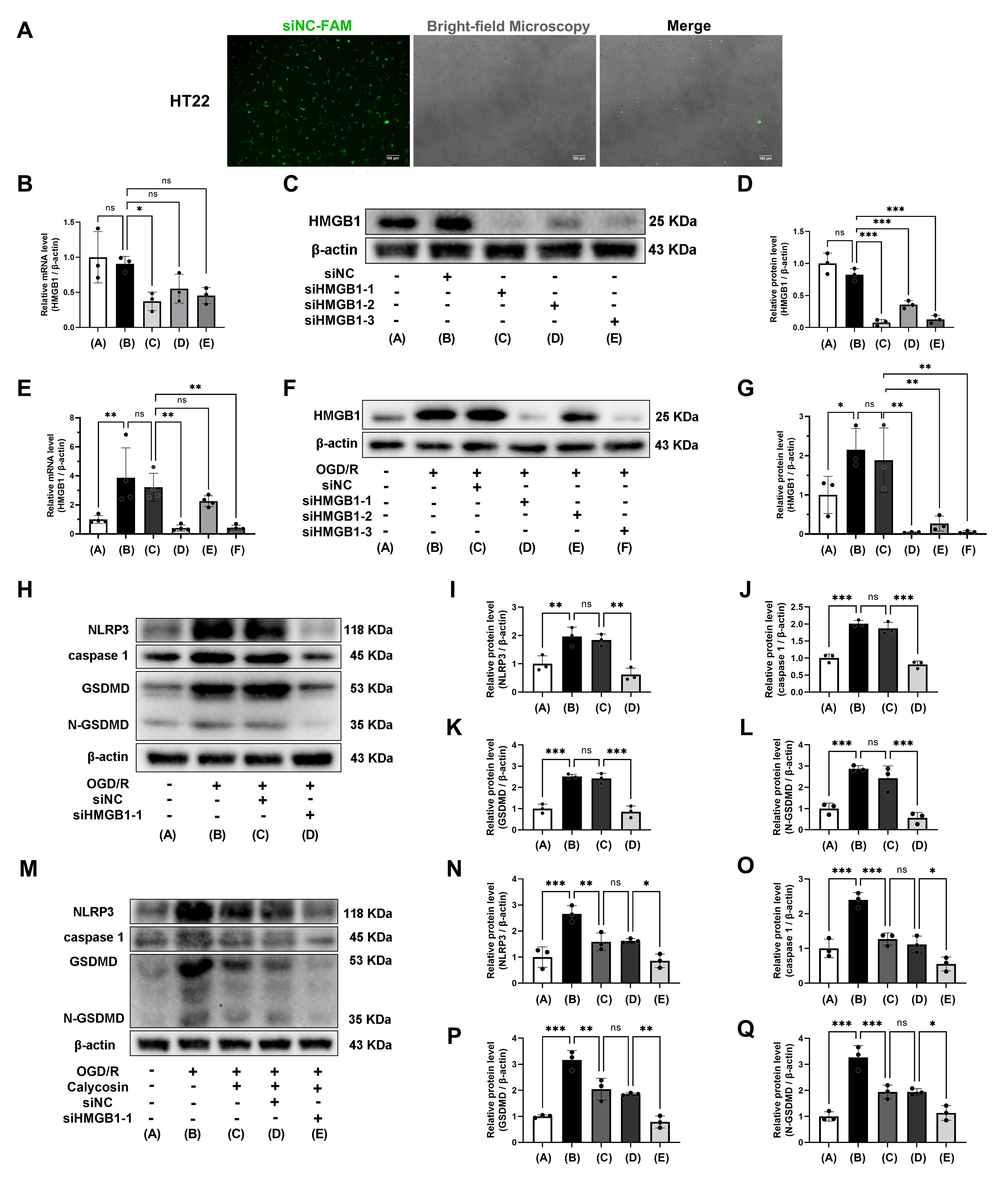

Supplement: Supplementary file 4 [file Supplementaryfile3.jpeg]

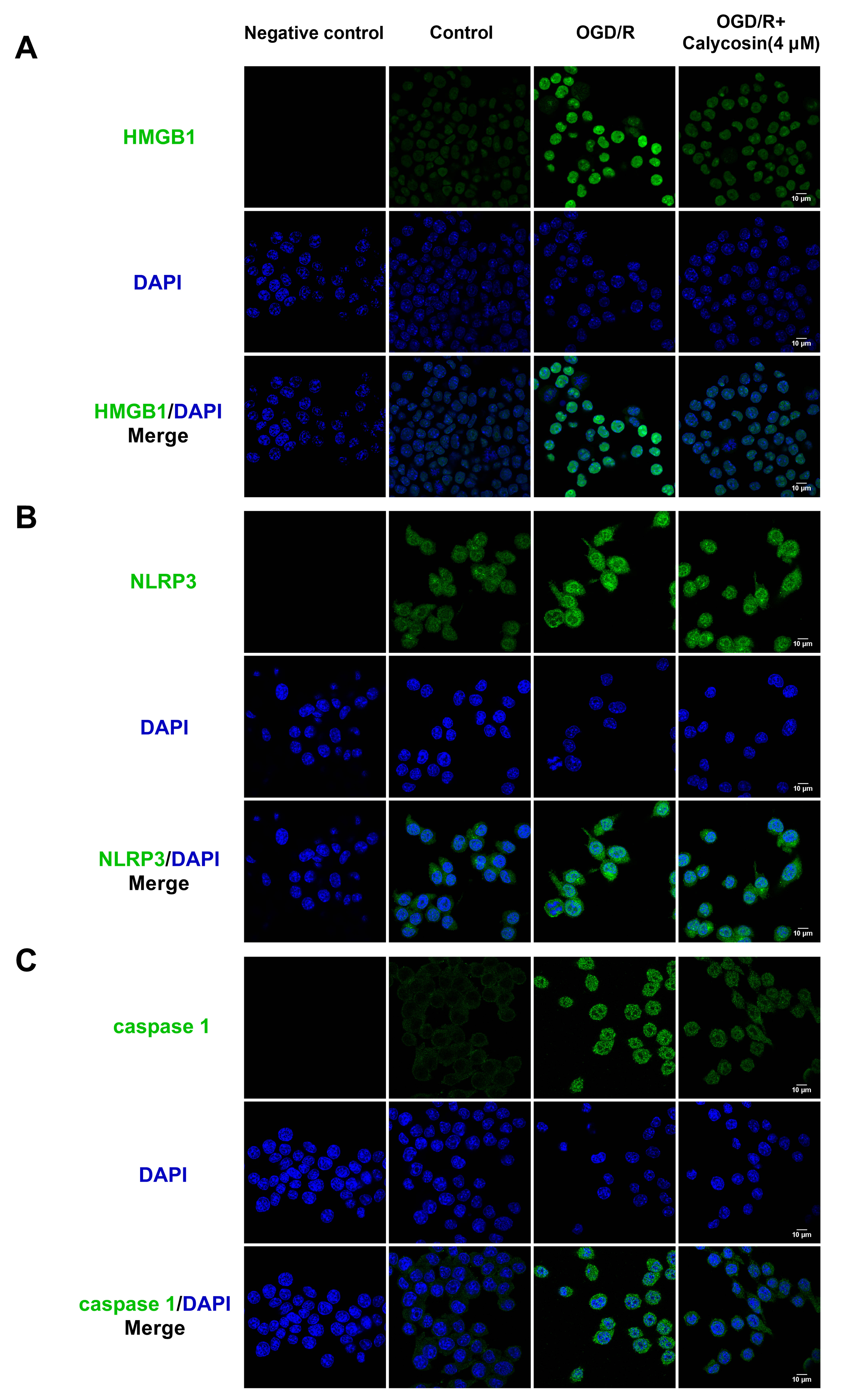

Supplement: Supplementary file 6 [file Supplementaryfile1.jpeg]
