# Supplementary material for: Intracerebroventricular calycosin attenuates cerebral ischemia-reperfusion injury in rats via HMGB1-dependent pyroptosis inhibition
Source: Front Pharmacol. 2025 Jun 18;16:1596087. doi: 10.3389/fphar.2025.1596087 (PMC12213575; doi:10.3389/fphar.2025.1596087)
Supplement: Supplementary file 2 [file Supplementaryfile6.docx]

Supplementary Figure S1.


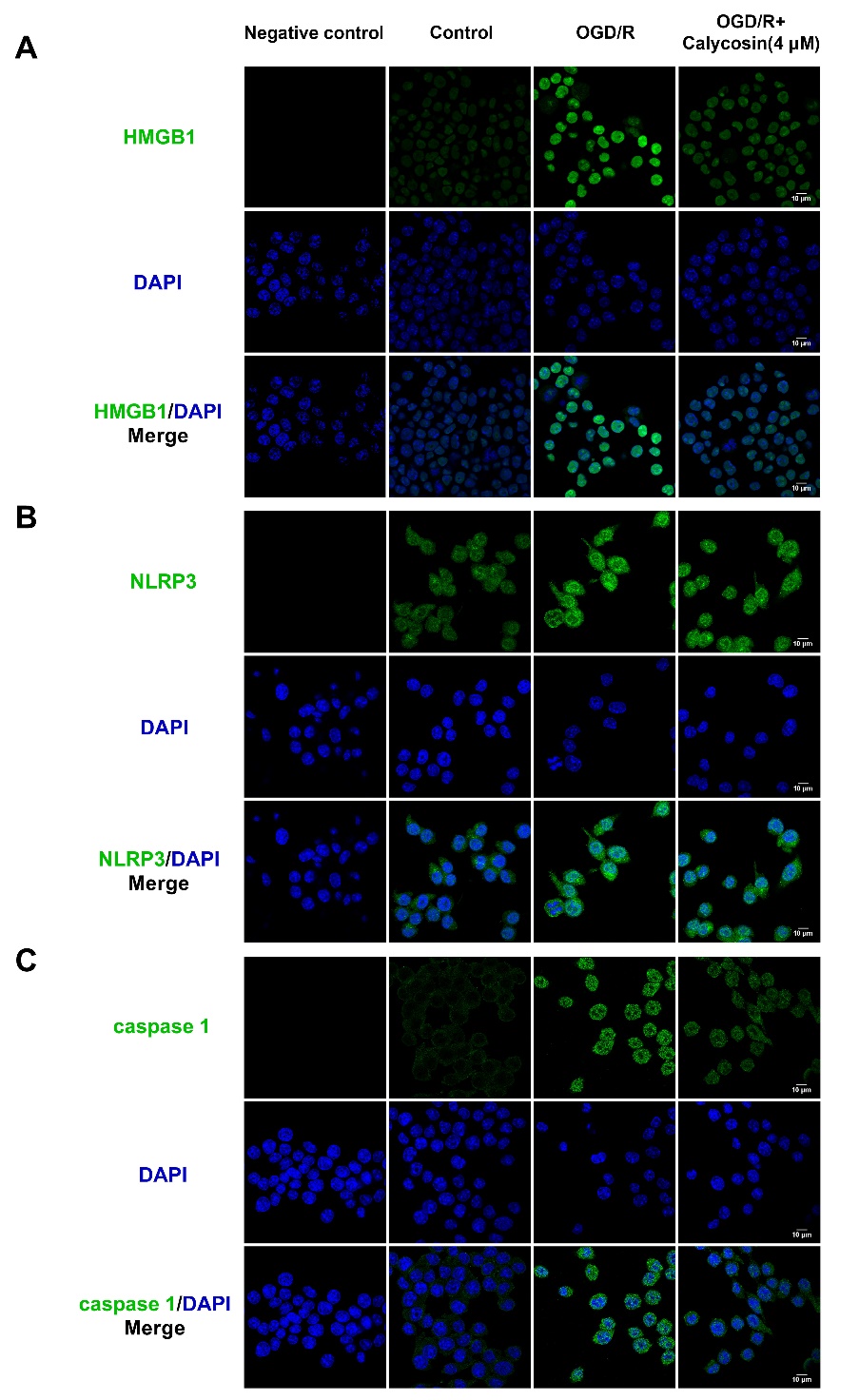


Figure S1. Fluorescence co-localization of (A)HMGB1 (green), (B)NLRP3 (green), (C)caspase 1 (green), and DAPI (blue) in HAPI cells following OGD/R exposure or calycosin treatment. Scale bar: 10 μm.

Supplementary Figure S2.


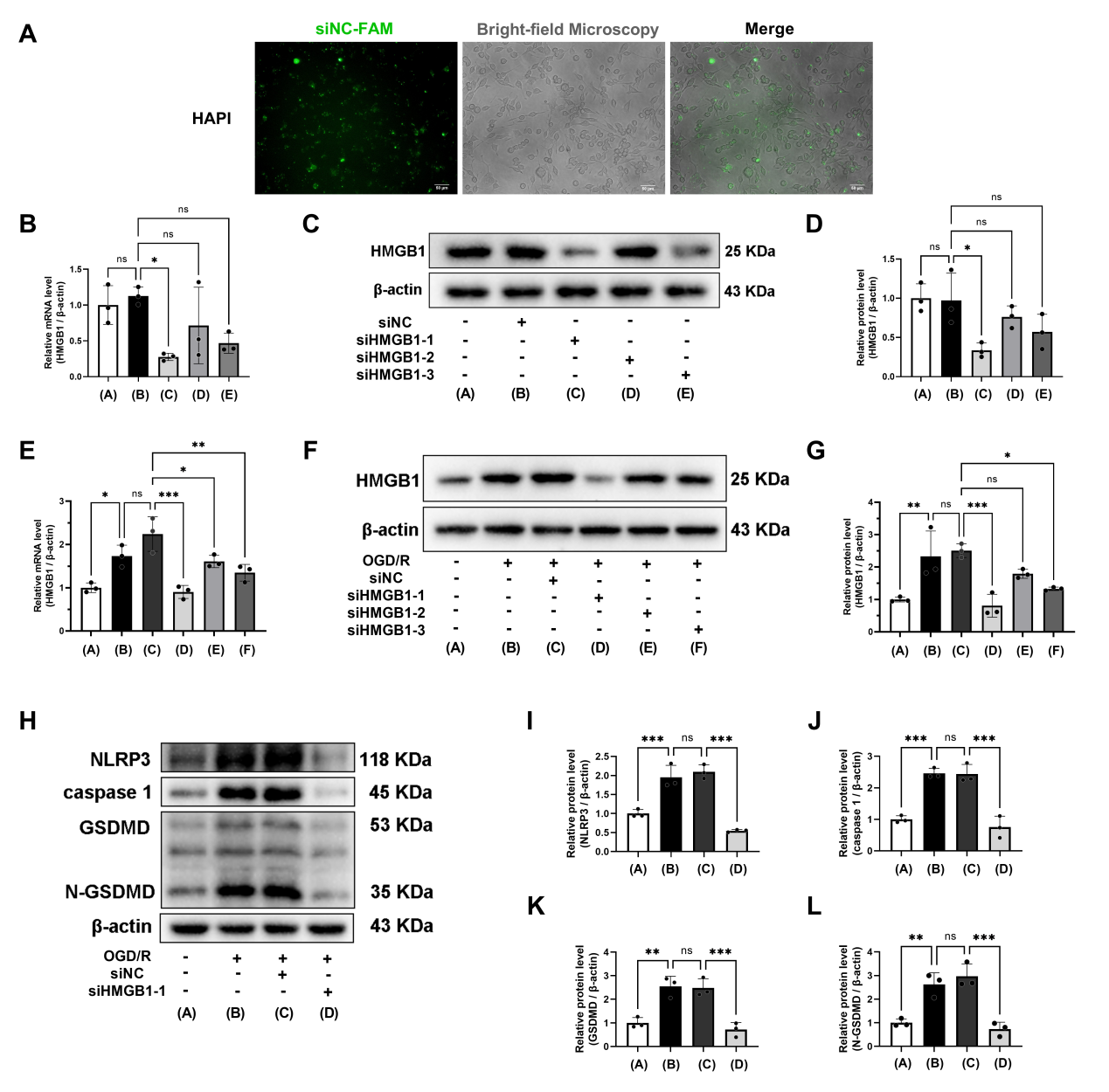


Figure S2. siHMGB1 significantly suppressed the upregulation of HMGB1-mediated pyroptosis markers in HAPI cells subjected to OGD/R injury. (A) Co-localization analysis of siNC-Fam-labeled immunofluorescence and Bright-field microscopy for evaluating transfection efficiency in HAPI cells. Scale bar: 50 μm. (B-D) Evaluation of siRNA transfection efficiency through quantitative analysis of HMGB1 mRNA and protein expression levels in HAPI cells. (E-G) Assessment of individual siRNA transfection efficacy through quantitative profiling of HMGB1 mRNA and protein expression in HAPI cells following OGD/R. (H-L) Western blot analysis of pyroptosis-related protein expression in HAPI cells post siHMGB1 transfection and OGD/R exposure. A β-actin internal control was employed. The mean ± SD is used to express the results. ***p<0.001, **p<0.01, and *p<0.05. n = 3 per group.

Supplementary Figure S3.


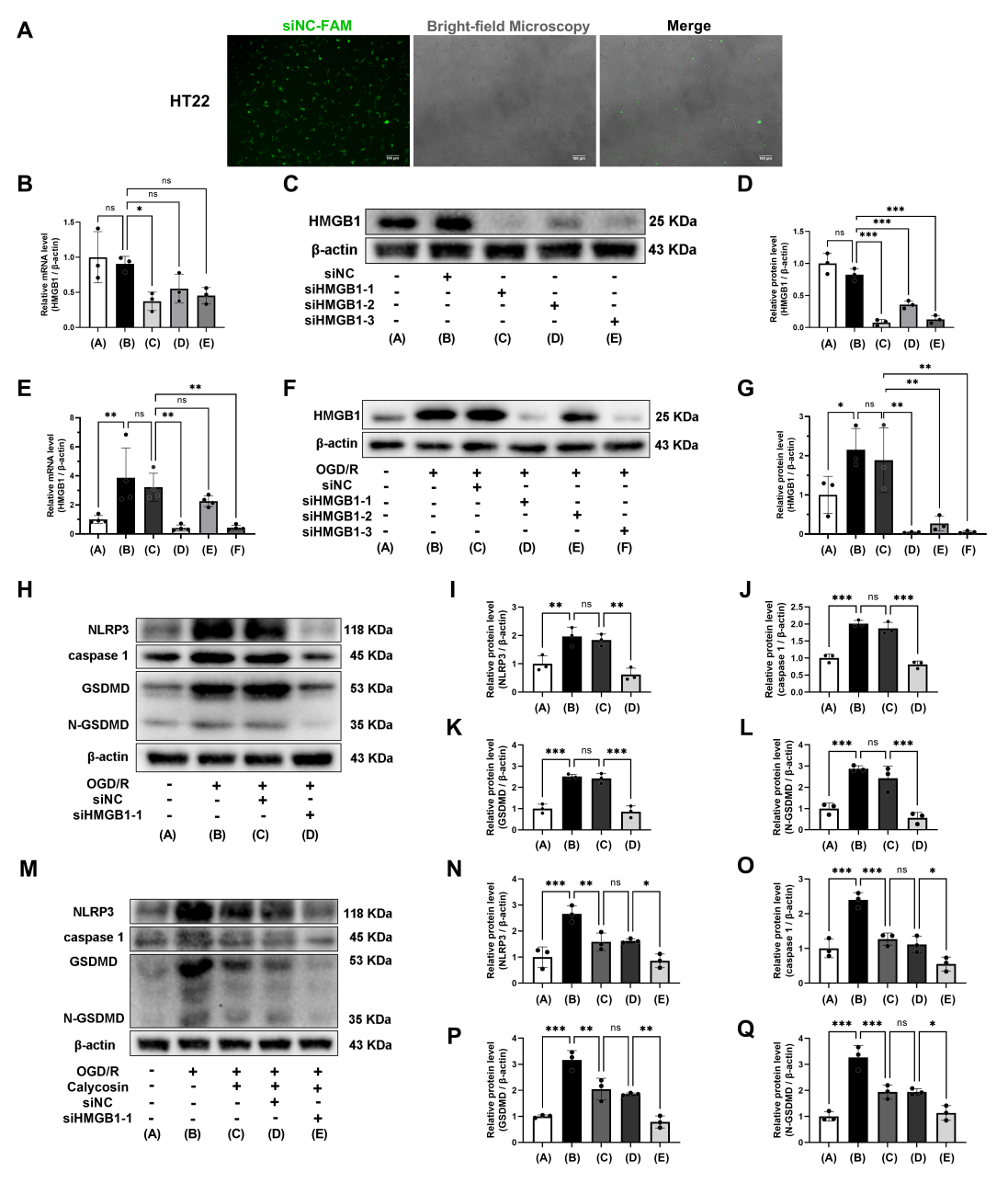


Figure S3. siHMGB1 significantly suppressed the upregulation of HMGB1-mediated pyroptosis markers in HT22 cells induced by OGD/R. (A) Assessment of HT22 cells transfection efficiency via siNC-FAM immunofluorescence/Bright-field image Co-localization. Scale bar: 100 μm. (B-D) Evaluation of siRNA transfection efficiency through quantitative analysis of HMGB1 mRNA and protein expression levels in HT22 cells. (E-G) Assessment of individual siRNA transfection efficacy through quantitative profiling of HMGB1 mRNA and protein expression in HT22 cells following OGD/R. (H-L) Western blot analysis of pyroptosis-related protein expression in HT22 cells post siHMGB1 transfection and OGD/R exposure. (M-Q) Calycosin effects on the pyroptosis marker proteins (NLRP3, caspase1, GSDMD, and N-GSDMD) in siHMGB1-transfected HT22 cells following OGD/R. A β-actin internal control was employed.The mean ± SD is used to express the results. ***p<0.001, **p<0.01, and *p<0.05. n = 3-4 per group.
