# Supplementary material for: Intracerebroventricular calycosin attenuates cerebral ischemia-reperfusion injury in rats via HMGB1-dependent pyroptosis inhibition
Source: Front Pharmacol. 2025 Jun 18;16:1596087. doi: 10.3389/fphar.2025.1596087 (PMC12213575; doi:10.3389/fphar.2025.1596087)
Supplement: Supplementary file 3 [file Supplementaryfile5.docx]

Supplementary Table S2. Summary of the qRT-PCR Primers sequences.

Table S2 qRT-PCR primer sequences

| Genes | | Primers （5′-3′） | Tm (℃) | Accession Number |
| --- | --- | --- | --- | --- |
| β-actin | F | CCCATCTATGAGGGTTACGC | 49.6 | NM_031144.3 |
|  | R | TTTAATGTCACGCACGATTTC | 50.9 |  |
| HMGB1 | F | AGGCTGACAAGGCTCGTTATG | 54.2 | NM_012963.4 |
|  | R | GATTTTGGGGCGGTACTCAG | 53.7 |  |
| NLRP3 | F | AGACCTCCAAGACCACGACTG | 56.1 | NM_001191642.1 |
|  | R | TTCCATCCGCAGCCAATGAAC | 54.2 |  |
| caspase 1 | F | CGGAGAGTCGGAGCTGATGTTG | 60.5 | NM_012762.3 |
|  | R | CTGGGCAGGCAGCAAATTCTTTC | 59.7 |  |
| IL-1β | F | GAAATGCCACCTTTTGACAGTG | 54.2 | NM_031512.2 |
|  | R | TGGATGCTCTCATCAGGACAG | 53.7 |  |
| IL-18 | F | GTGAACCCCAGACCAGACTG | 53.7 | NM_019165.2 |
|  | R | CCTGGAACACGTTTCTGAAAGA | 54.2 |  |
