# Supplementary material for: Intracerebroventricular calycosin attenuates cerebral ischemia-reperfusion injury in rats via HMGB1-dependent pyroptosis inhibition
Source: Front Pharmacol. 2025 Jun 18;16:1596087. doi: 10.3389/fphar.2025.1596087 (PMC12213575; doi:10.3389/fphar.2025.1596087)
Supplement: Supplementary file 5 [file Supplementaryfile4.docx]

Supplementary Table S1. Summary of the siRNAs sequences.

Table S1 Negative control and anti HMGB1 siRNAs sequences

| Gene | siRNA | Sequences |
| --- | --- | --- |
| Negative control | siNC | Sense 5’-UUCUCCGAACGUGUCACGUdTdT-3’ |
|  |  | Antisense 5’-ACGUGACACGUUCGGAGAAdTdT-3’ |
| HMGB1 | siRNA-1 | Sense 5’-CGGAGGAAAAUCAACUAAACAUGTT-3’ |
|  |  | Antisense 5’-CAUGUUUAGUUGAUUUUCCUCCGTT-3’ |
|  | siRNA-2 | Sense 5’-GAGGAGGAAUACUGAACAUTT-3’ |
|  |  | Antisense 5’-AUGUUCAGUAUUCCUCCUCTT-3’ |
|  | siRNA-3 | Sense 5’-GGAGGAAGACGAAGAUGAATT-3’ |
|  |  | Antisense 5’-UUCAUCUUCGUCUUCCUCCTT-3’ |
